# Statin use in pregnancy and risk of congenital malformations: a Norwegian nationwide study

**DOI:** 10.1093/eurheartj/ehaf592

**Published:** 2025-08-21

**Authors:** Jacob J Christensen, Kirsten B Holven, Martin P Bogsrud, Kjetil Retterstøl, Jeanine E Roeters van Lennep, Trond M Michelsen, Marit B Veierød, Hedvig Nordeng

**Affiliations:** Department of Nutrition, Institute of Basic Medical Sciences, University of Oslo, P.O. Box 1046 Blindern, Oslo 0317, Norway; Department of Nutrition, Institute of Basic Medical Sciences, University of Oslo, P.O. Box 1046 Blindern, Oslo 0317, Norway; The National Advisory Unit on Familial Hypercholesterolemia, Oslo University Hospital, Oslo, Norway; Unit for Cardiac and Cardiovascular Genetics, Oslo University Hospital, Oslo, Norway; Department of Nutrition, Institute of Basic Medical Sciences, University of Oslo, P.O. Box 1046 Blindern, Oslo 0317, Norway; The Lipid Clinic, Oslo University Hospital, Oslo, Norway; Department of Internal Medicine, Erasmus MC Cardiovascular Institute, Erasmus MC University Medical Center, Rotterdam, the Netherlands; Department of Obstetrics, Division of Obstetrics and Gynecology, Oslo University Hospital, Oslo, Norway; Faculty of Medicine, University of Oslo, Oslo, Norway; Oslo Centre for Biostatistics and Epidemiology, Department of Biostatistics, Institute of Basic Medical Sciences, University of Oslo, Oslo, Norway; PharmacoEpidemiology and Drug Safety Research Group, University of Oslo, Oslo, Norway; Department of Child Health and Development, Norwegian Institute of Public Health, Oslo, Norway

**Keywords:** Statins, Drug safety, Pregnancy, Congenital malformations, Nationwide, Registry

## Abstract

**Background and Aims:**

Statins and other lipid-modifying agents (LMAs) have traditionally been contraindicated during pregnancy due to concerns about harmful fetal effects; however, the risks associated with exposure to statins and other LMAs in human pregnancies remain unclear. Therefore, this study aimed to examine the associations between statin and LMA exposure in pregnancy and congenital malformations in offspring, while updating a 2022 meta-analysis with the results from the present study.

**Methods:**

National registry data were linked for all pregnant women in Norway in 2005–18. Associations between first-trimester statin prescription fills and congenital malformations were estimated with mixed-effects logistic regression, adjusting for age, parity, pre-pregnancy folate use, smoking in early pregnancy, comorbidity, and co-medication. Meta-analyses were performed with the generic inverse–variance method and random-effects model.

**Results:**

Congenital malformations occurred among 34 755 out of 803 830 (4.3%) statin non-exposed pregnancies, 74 out of 1255 (5.9%) statin-discontinuer pregnancies, and 19 out of 283 (6.7%) statin-exposed pregnancies. Adjusted odds ratios (ORs) for exposed vs non-exposed pregnancies were 1.30 [95% confidence interval (CI) .81–2.09] for any, 1.15 (.61–2.19) for major, and 1.47 (.75–2.89) for minor malformations. In analyses of exposed vs discontinuer pregnancies, there were no associations between statin exposure and any (adjusted OR 1.01, 95% CI .59–1.72), major (1.08, .52–2.25), or minor malformations (.94, .44–2.00). Results were similar across sensitivity analyses. There was also no association between any LMA exposure and heart malformations (adjusted OR 1.22, 95% CI .50–3.01). The updated meta-analysis suggested no increased risk of major (adjusted OR 1.06, 95% CI .86–1.31) or heart malformations (1.24, 95% CI .94–1.64).

**Conclusions:**

In this large, nationwide study and updated meta-analysis, no significant association was observed between first-trimester exposure to statins or other LMAs and congenital malformations. Although limited power may have prevented detection of weak but clinically relevant associations, the findings do not support a strong or independent association between statin exposure in pregnancy and congenital malformations.

## Structured Graphical Abstract

**Structured Graphical Abstract:**
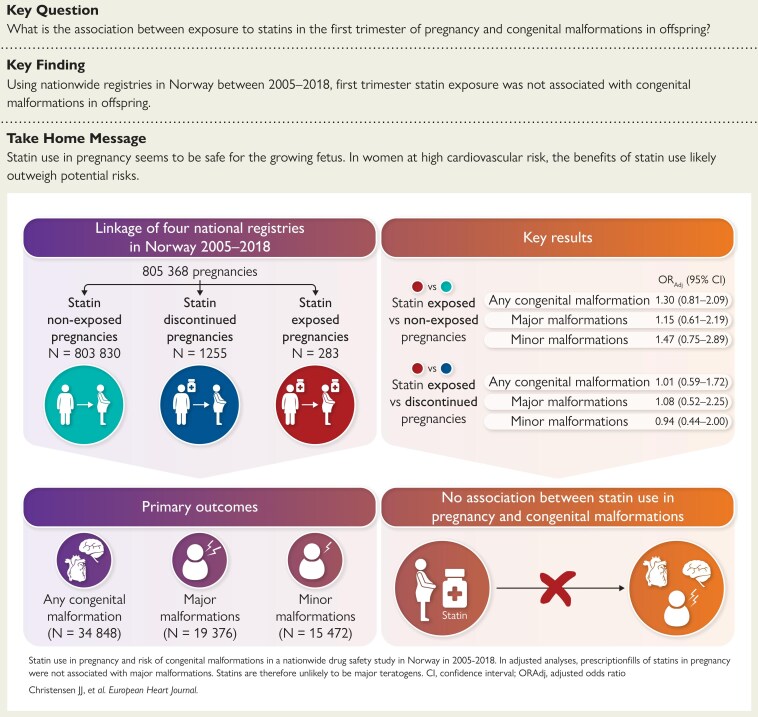

**See the editorial comment for this article ‘Statin therapy during pregnancy’, by P.E. Stürzebecher and U. Laufs, https://doi.org/10.1093/eurheartj/ehaf666.**

## Introduction

Statins have traditionally been contraindicated during pregnancy and lactation due to concerns about their potential risks.^1^ However, the effects of statins and other lipid-modifying agents (LMAs) on human pregnancies have remained unclear.^2^ Some LMAs could potentially affect placental development, impair fetal growth, and lead to structural fetal anomalies, while cholesterol is vital for embryonic development. Early animal studies suggested teratogenic effects of statins at high doses, yet human data have been scarce^2^; therefore, guidelines recommend most women planning pregnancy to discontinue statin therapy.^1^

In a 2021 review, the Food and Drug Administration (FDA) examined the relationship between statin exposure in pregnancy and health outcomes, especially major congenital malformations and miscarriage.^3^ They concluded that robust observational studies in humans did not support results from animal studies. In a recent meta-analysis summarizing five cohort studies^4–9^ and one nested case control study,^10^ Karadas *et al.*^11^ concluded that first-trimester statin exposure was not associated with major congenital malformations [summary odds ratio (OR) 1.05, 95% confidence interval (CI) .84–1.31] or heart defects (OR 1.24, 95% CI .93–1.66). Vahedian-Azimi *et al.*^12^ reached comparable conclusions despite more heterogeneous inclusion criteria, including studies with fewer exposed pregnancies and varied exposure windows.

Based on the available evidence, the FDA requested removal of the strongest warning against using statins in pregnancy, while still advising most pregnant patients to stop taking statins.^3^ They noted that multiple studies found no increased risk of major birth defects following statin use in pregnancy.^3^ Due to its size and methodological rigour, the core evidence was the cohort study by Bateman *et al.*^4^; in this study, using linkage data from Medicaid of 1152 statin-exposed pregnant women compared to 885 844 non-exposed women, the adjusted relative risk of congenital malformations in the exposed vs non-exposed group was 1.07 (95% CI .85–1.37). The FDA emphasized the need for additional drug safety analyses with detailed and objective information on exposures, outcomes, and confounders, including relevant co-medication.^3^

With this background, we assessed the association between the use of statins and other LMAs in pregnancy and the risk of congenital malformations in the offspring. In addition, we updated the summary estimates from the most recent meta-analysis^11^ with the estimates from the present study.

## Methods

### Study design, data sources, and study population

The present study is based on the cohort thoroughly described in our recent study on drug utilization.^13^ Individual-level data were linked from four national health registries in Norway from 2005 until 2018: the Medical Birth Registry of Norway (MBRN), the Norwegian Prescription Registry (NorPD), the Norwegian Patient Registry (NPR), and the Norway Control and Payment of Health Reimbursement Database (KUHR). The MBRN registers pregnancy-related information for all pregnancies in Norway ending after gestational week 12, including offspring health from birth until 1 year of age. NorPD records all prescribed medications dispensed at Norwegian pharmacies to non-institutionalized individuals, and medications are classified according to the WHO’s Anatomical Therapeutic Chemical (ATC) Classification System. NPR includes information on activity in both secondary and tertiary health care settings in Norway and uses the International Classification of Diseases, Tenth Revision (ICD-10). KUHR stores information on activity in primary healthcare settings in Norway. KUHR records the reason for the utilization of health care services and uses both ICD-10 and the International Classification of Primary Care-2 (ICPC-2).

We included all women with a pregnancy recorded in the MBRN in 2005–18. Pregnancies with missing gestational length were excluded, because the timing of drug exposure could not be calculated for those pregnancies. Multiple birth pregnancies and pregnancies with offspring with chromosomal abnormalities were also excluded, because these conditions were expected to affect the risk of malformations.

This project was approved by the Regional Committee for Research Ethics in South-Eastern Norway (approval number 2018/140/REK South-East) and by the Data Protection Officer at the University of Oslo (approval number 580338033).

### Data measurements and variables

#### Exposure variables

Pregnancies among women using statins or other LMAs were identified through dispensed prescriptions in NorPD. Exposure was defined as at least one prescription fill within specific time frames across pregnancy, based on the date of prescription dispensing. The groups of interest were defined as follows: the *exposed* group, including pregnancies with prescription fills during the first trimester (from conception to 93 days, the critical period for the development of major congenital malformations); the *discontinuer* group, including pregnancies with prescription fills up to 1 year before conception but not during the first trimester; and the *non-exposed* group, including pregnancies with no prescription fills during the first trimester or in the year before conception (see Supplementary data online, *Figure S1A*). The latter group could have filled prescriptions prior to this, or any time after the first trimester.

We assumed that all the dispensed LMAs were consumed as 1 DDD per day. The main exposures of interest were prescription fills of *statin* LMAs (statin monotherapy and statin combination therapy) and *any* LMA (monotherapy or combination therapy) (see Supplementary Methods, Supplementary data online, *Table S1*). Any LMA includes both statin and non-statin LMA drugs. Isolated use of non-statin LMAs had low prevalence in the present study population and was not used in analyses.^13^

The main exposure window was first-trimester prescription fills because organogenesis and thus most major malformations occur in gestational weeks 3–8. In additional analyses, we examined *carryover exposure*, defined as first-trimester prescription fills *or* prescription fills before conception with package durations estimated to carry over into the first trimester (see Supplementary data online, *Figure S1B*). Carryover analyses were considered relevant since women often do not recognize they are pregnant until 4–6 weeks into pregnancy^14,15^; this decision was supported by the shape of the LMA prescription filling histogram across pregnancy, as previously reported^13^ and shown in Supplementary data online, *Figure S1*.

#### Outcome variables

Congenital malformations were derived from MBRN and coded as binary variables (yes/no). *Any congenital malformation* was defined as at least one major or minor malformation in any organ system, and ICD-10 codes were derived from the EUROCAT subgroups of congenital anomalies (Version 2014, Guide 1.4, Chapter 3.3) (see Supplementary Methods, Supplementary data online, *Table S2*).^16^  *Minor malformations* were defined as *isolated* minor malformations in any organ system, that is, ‘structural changes that pose no significant health problem in the neonatal period and tend to have limited social or cosmetic consequences for the affected individual’^17^; ICD-10 codes were derived from the EUROCAT exclusion list for minor malformations (Version 2014, Guide 1.4, Chapter 3.2) (see Supplementary Methods, Supplementary data online, *Table S3*).^16^  *Major malformations* were defined as ‘structural changes in any organ system that have significant medical, social or cosmetic consequences, and typically require medical intervention’^17^ and were derived by excluding isolated minor malformations from the *Any congenital malformation* variable, as per EUROCAT standard (see Supplementary Methods, Supplementary data online, *Table S2*).^16^  *Heart malformations* are a subgroup of major malformations and were defined as severe congenital heart defects but not benign conditions, e.g. related to premature birth (see Supplementary Methods, Supplementary data online, *Table S2*).

#### Covariates

From the MBRN, we derived age (≤24/25–34/≥35 years), parity (nulliparous/other), pre-pregnancy folate use (yes/no), and smoking in early pregnancy (yes/no/missing). From NPR and KUHR, we derived comorbidities registered any time before conception, including pre-pregnancy diabetes mellitus, pre-pregnancy hypertension, and pre-pregnancy cardiovascular disease (CVD) (see Supplementary Methods). From NorPD, we derived co-medication registered 6–12 months before conception, including pre-pregnancy use of diabetes drugs, pre-pregnancy use of anti-thrombotic agents, and pre-pregnancy use of CVD drugs (see Supplementary Methods).

Two indices based on the included comorbidities (the comorbidity index) or co-medications (the co-medication index or comorbidity severity index) were also derived by summing the number of groups of diseases or medications that any single pregnancy was exposed to before conception, and then collapsing to three levels (0/1/≥2).

### Statistical analyses

Descriptive results are presented as frequencies (%) or medians [interquartile ranges (IQR)].

The unit of analysis was the pregnancy, and ∼50% of the women had more than one pregnancy in 2005–18. Mixed-effects logistic regression was performed for each exposure–outcome combination with a random intercept for a maternal identifier (glmer function in the lme4 package in R).

Covariates were included in the models based on assumptions in directed acyclic graphs (see Supplementary data online, *Figure S2*),^18^ aiming to account especially for confounding by indication. In the main analyses, adjustments were made for age, parity, pre-pregnancy folate use, smoking in early pregnancy, the comorbidity index, and the co-medication (comorbidity severity) index (model 3b in Supplementary data online, *Figure S3*). The comorbidity and co-medication indices were included in the main adjusted analyses to limit the number of terms in our models; however, in sensitivity analyses, we instead adjusted for pre-pregnancy diagnoses of diabetes mellitus, hypertension, or CVD and pre-pregnancy use of medications for diabetes mellitus, hypertension, or CVD (model 4b in Supplementary data online, *Figure S3*).

In the main analyses, statin-discontinuer and statin-exposed pregnancies were compared to non-exposed pregnancies. In secondary analysis, we restricted the analyses to the discontinuer and exposed pregnancies and used discontinuer pregnancies as the reference, since they more accurately consider confounding by indications and thus most accurately account for background risk of the pregnancies. Heart malformations were examined for any LMA exposure only, as the number of events for statin exposure was low (<3).

Smoking in early pregnancy was the only variable with missing values, and missingness was related to both exposure and outcome (data not shown). We assumed missing not at random (MNAR), and multiple imputation was therefore not used. However, other strategies were explored, including (i) complete case analyses, (ii) setting missing entries to ‘no smoking’, and (iii) setting missing entries as a separate category ‘missing’ (reported in results).

Several sensitivity analyses were conducted. First, as mentioned previously, we examined exposure to *any LMA* to explore the combined effects of statins and non-statin LMAs, and we examined carryover exposure to explore discontinuation misclassification. Second, we examined lipophilic statins only (simvastatin, lovastatin, fluvastatin, atorvastatin, and cerivastatin), as these have been suggested to have a greater teratogenic potential compared to hydrophilic statins. Third, pregnancies exposed to non-LMA co-medication in the first trimester were excluded to focus on isolated statin exposure (see Supplementary Methods, Supplementary data online, *Table S1*). And fourth, pregnancies exposed to non-statin LMAs in the first trimester were excluded to further eliminate potential harmful effects by co-medication (see Supplementary Methods, Supplementary data online, *Table S4*).

Finally, the summary estimates in the meta-analysis of Karadas *et al.*^11^ for major congenital malformations and heart malformations were updated by adding the estimates from the present study (exposed vs non-exposed pregnancies). To ensure that we used the same method as Karadas *et al.*^11^ and that the updated pooled estimates differ because of the addition of our estimates, we first reproduced their results (data not shown) and then performed the analyses adding our result. For meta-analysis of crude estimates, the metabin function in the meta package in R was used to calculate the OR and 95% CIs for each study by specifying events and sample sizes from *Figures 2* and *4* (major and heart malformations, respectively) in Karadas *et al.*^11,19^ To combine the adjusted estimates, the metagen function in the meta package in R was applied, by specifying log OR and standard errors (SE) from *Figures 3* and *5* (major and heart malformations, respectively) in Karadas *et al.*^11^ Random-effects meta-analysis was performed for crude and adjusted estimates with the inverse–variance method and the DerSimonian–Laird approach for estimating tau^2^. Heterogeneity between studies was tested with the *Q*-test, and the extent of heterogeneity was quantified by *I*^2^. Forest plots were created using the forest function in the meta package in R.

All data analyses were performed in R version 3.6.2^20^ using RStudio (Boston, MA, USA).

## Results

**Figure 1:**
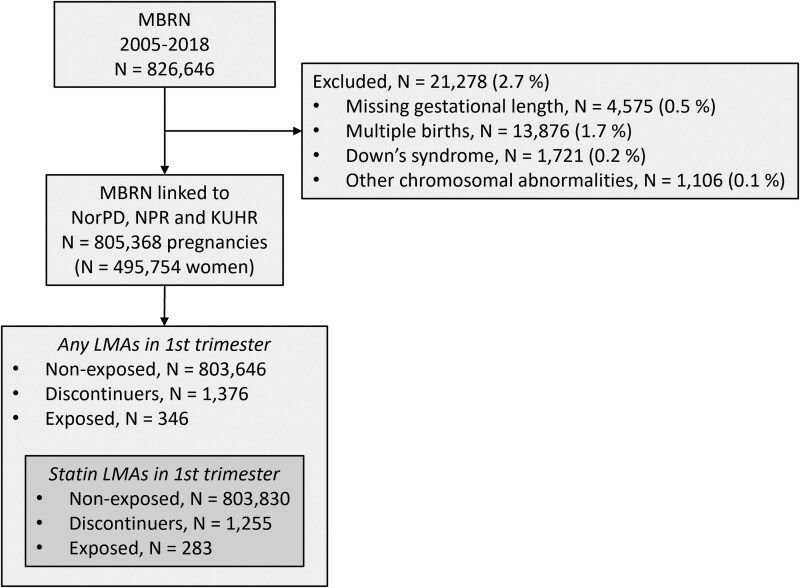
Flow chart. We included all women with a pregnancy recorded in the MBRN in 2005–18 and then linked these to NorPD, NPR, and KUHR. See Methods for definitions of non-exposed, discontinuer, and exposed groups. KUHR, Norway Control and Payment of Health Reimbursement Database; LMA, lipid-modifying agent; MBRN, Medical Birth Registry of Norway; NorPD, Norwegian Prescription Registry; NPR, Norwegian Patient Registry

We included 805 368 pregnancies for 495 754 unique women (*Figure 1*; see Supplementary data online, *Figure S4* for carryover exposure). Compared to the non-exposed pregnancies (*n* = 803 830), statin-discontinuer (*n* = 1255) and statin-exposed (*n* = 283) pregnancies had higher maternal age (medians 30, 31, and 33 years, respectively), pre-pregnancy maternal weight (medians 65, 70, and 72 kg, respectively), prevalence of smoking in early pregnancy (8.4%, 12%, and 16%, respectively), comorbidity index (.2%, 5.2%, and 12% with ≥2 comorbidities, respectively), and co-medication index (.1%, 6.8%, and 12% with ≥2 co-medications, respectively) (*Table 1*; see Supplementary data online, *Table S5* for detailed data on indications for therapy, co-medication, and comorbidity). Indications for therapy were established atherosclerotic CVD (ASCVD), high risk of ASCVD, or familial hypercholesterolaemia (FH) or other dyslipidaemias (see Supplementary data online, *Table S5*). Simvastatin and atorvastatin were the most used statin type (see Supplementary data online, *Table S6*). Among exposed pregnancies, simvastatin was predominantly prescribed at 20 mg (36%) and 40 mg (27%), with a median of six prescription fills (25th–75th percentiles: 3–11) (see Supplementary data online, *Table S7*).

**Table 1:** Characteristics of pregnancies exposed or not exposed to statins in the first trimester

	Non-exposed pregnancies, *N* = 803 830	Discontinuer pregnancies, *N* = 1255	Exposed pregnancies, *N* = 283
*Descriptives* ^a,b,c^			
Age, years, median (IQR)	30 (26–33)	31 (28–35)	33 (29–38)
Age, years			
≤24	122 115 (15)	99 (7.9)	26 (9.2)
25–34	526 739 (66)	776 (62)	142 (50)
≥35	154 976 (19)	380 (30)	115 (41)
Employed	520 509 (81)	874 (85)	175 (75)
Registered partner/cohabitant	745 962 (93)	1149 (92)	254 (90)
Nulliparous	339 272 (42)	592 (47)	131 (46)
Previous pregnancy loss	178 752 (24)	289 (24)	56 (21)
Pre-pregnancy weight, kg	65 (58–75)	70 (60–84)	72 (62–85)
Pre-pregnancy folate use	227 410 (28)	460 (37)	75 (27)
Smoking in early pregnancy			
No	624 044 (78)	966 (77)	208 (73)
Yes	67 152 (8.4)	147 (12)	44 (16)
Missing	112 634 (14)	142 (11)	31 (11)
Liveborn infant	796 873 (99.1)	1240 (98.8)	281 (99.3)
*Comorbidities* ^d^			
Pre-pregnancy diabetes mellitus	5775 (.7)	162 (13)	51 (18)
Pre-pregnancy hypertension	11 357 (1.4)	117 (9.3)	47 (17)
Pre-pregnancy other CV diagnoses	21 146 (2.6)	147 (12)	55 (19)
Comorbidity index			
0	767 312 (95)	896 (71)	169 (60)
1	34 809 (4.3)	294 (23)	79 (28)
≥ 2	1709 (.2)	65 (5.2)	35 (12)
*Co-medication* ^e^			
Pre-pregnancy use of diabetes drugs	5852 (.7)	144 (11)	45 (16)
Pre-pregnancy use of anti-thrombotic agents	4714 (.6)	107 (8.5)	30 (11)
Pre-pregnancy use of CVD drugs	14 957 (1.9)	171 (14)	64 (23)
Co-medication index			
0	779 235 (97)	923 (74)	180 (64)
1	23 689 (2.9)	247 (20)	70 (25)
≥ 2	906 (.1)	85 (6.8)	33 (12)

CV, cardiovascular; CVD, cardiovascular disease; IQR, interquartile range; KUHR, Norway Control and Payment of Health Reimbursement Database; MBRN, Medical Birth Registry of Norway; NorPD, Norwegian Prescription Registry; NPR, Norwegian Patient Registry.

^a^ *n* (%) for all variables except for pre-pregnancy weight (median and 25th–75th percentile).

^b^ *n* = 644 613 for employed, *n* = 757 492 for previous pregnancy loss, and *n* = 413 420 for pre-pregnancy weight; for other variables, *n* = 805 368. Because smoking was included in the regression models, we set missing values to a separate level indicating missingness.

^c^Derived from MBRN.

^d^Derived from NPR and KUHR.

^e^Derived from NorPD.

### Analyses with non-exposed pregnancies as the reference group

**Figure 2:**
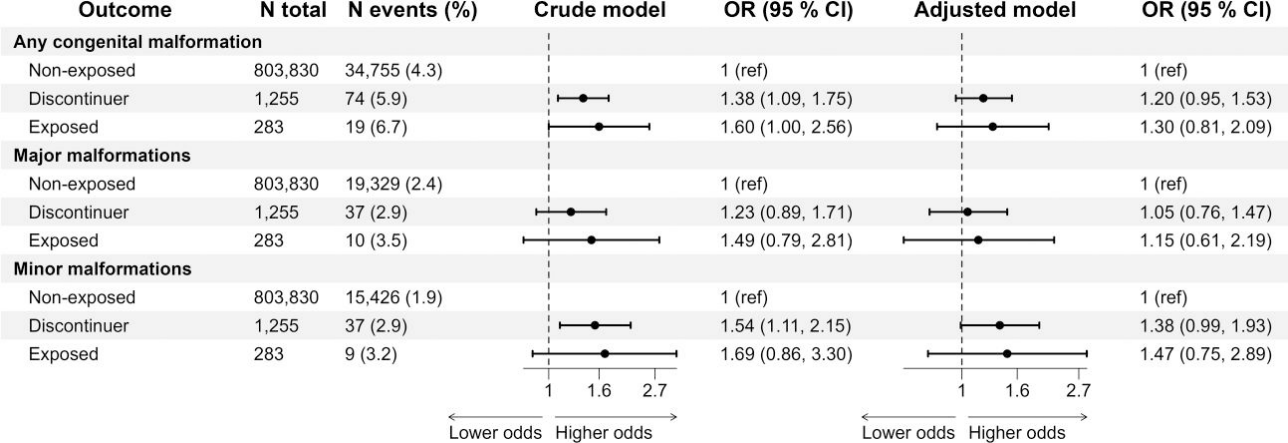
Associations between statin exposure and congenital malformations. The exposed group had prescription fills in the first trimester, while discontinuers had prescription fills up to 1 year before conception, but not during the first trimester; and non-exposed had no prescription fills during the first trimester and up to 1 year before conception. Adjustments were made for age, parity, pre-pregnancy folate use, early pregnancy smoking, comorbidity index, and comorbidity severity (co-medication) index

Congenital malformations occurred among 34 755 out of 803 830 (4.3%) statin non-exposed pregnancies, 74 out of 1255 (5.9%) statin-discontinuer pregnancies, and 19 out of 283 (6.7%) statin-exposed pregnancies (*Figure 2*). The OR estimates were attenuated and not significant in the adjusted analyses: adjusted ORs (95% CI) for exposed vs non-exposed pregnancies were 1.30 (.81–2.09) for any, 1.15 (.61–2.19) for major, and 1.47 (.75–2.89) for minor malformations (*Figure 2*; Supplementary data online, *Figure S3* for all adjustment levels).

The 46 cases of minor malformations among discontinuers and exposed pregnancies had mainly tongue tie or cyst of tongue (*n* = 17); patent ductus arteriosus due to preterm birth (*n* = 10); clicking hip, subluxation, or unstable hip (*n* = 5); undescended testicle (*n* = 4); and deformities of the feet (*n* = 3), as well as fewer cases of miscellaneous other conditions.

Results were similar in all sensitivity analyses, including using prescription fill of any LMA (see Supplementary data online, *Figure S5*), first-trimester carryover exposure (see Supplementary data online, *Figure S6* for statins and Supplementary data online, *Figure S7* for any LMA), excluding pregnancies exposed to non-statin LMAs in first trimester (see Supplementary data online, *Figure S8*), excluding pregnancies exposed to non-LMA medications in first trimester (see Supplementary data online, *Figure S9*), and including lipophilic statins only (see Supplementary data online, *Figure S10*). Also, we found no association between exposure to any LMA and heart malformations, with an adjusted OR of 1.22 (95% CI.50–3.01) (see Supplementary data online, *Figures S5*  *and S7* for carryover exposure).

### Analyses with discontinuer pregnancies as the reference group

**Figure 3:**
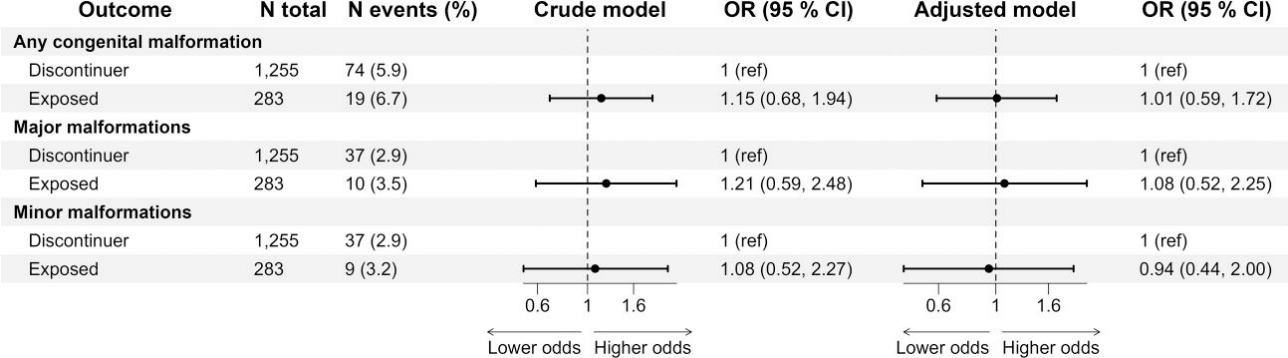
Associations between statin exposure and congenital malformations among statin continuers vs discontinuers (reference group). The exposed group had prescription fills in the first trimester, while discontinuers had prescription fills up to 1 year before conception, but not during the first trimester. Adjustments were made for age, parity, pre-pregnancy folate use, early pregnancy smoking, comorbidity index, and comorbidity severity (co-medication) index

In the analyses restricted to exposed vs discontinuer pregnancies, no associations were found with any (adjusted OR 1.01, 95% CI .59–1.72), major (1.08, 95% CI .52–2.25), or minor malformations (.94, 95% CI .44–2.00) (*Figure 3*). Results were similar in sensitivity analyses using prescription fill of any LMA (see Supplementary data online, *Figure S11*) and first-trimester carryover exposure (see Supplementary data online, *Figure S12* for statins and Supplementary data online, *Figure S13* for any LMA). Moreover, there was no association between exposure to any LMA (exposed vs discontinuer pregnancies) and heart malformations (see Supplementary data online, *Figures S7*  *and S9* for carryover exposure).

### Updated meta-analysis

**Figure 4:**
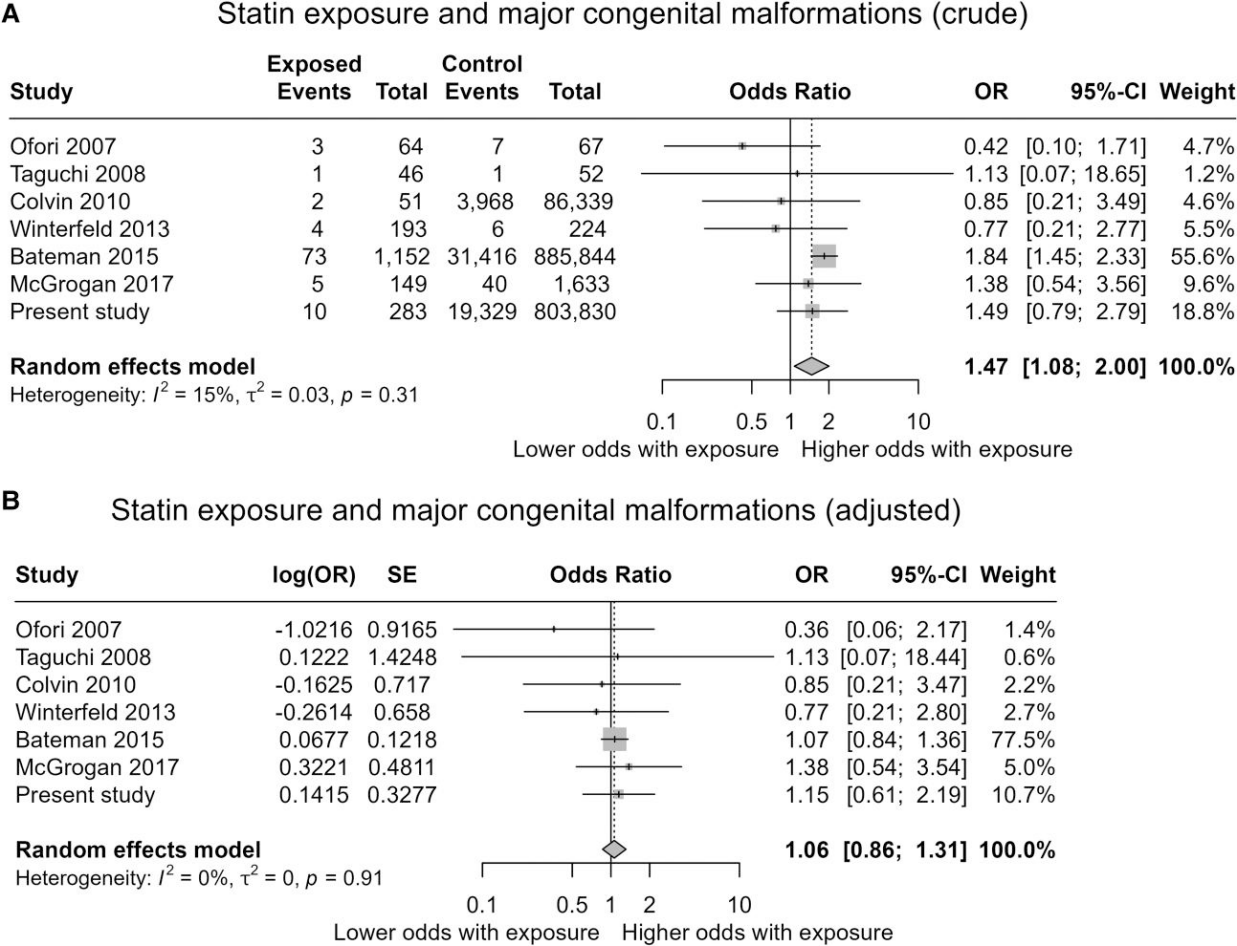
Meta-analysis of studies on statin exposure and major congenital malformations. Ofori 2007 was a nested case–control study, while the other studies were cohort studies. Both crude (*A*) and adjusted (*B*) estimates are shown. SE, standard error

Finally, we updated the meta-analysis by Karadas *et al.*^11^ of statin exposure and major congenital malformations (*Figure 4*) and any LMA exposure and heart malformations (see Supplementary data online, *Figure S14*) with the estimates from the present study (exposed vs non-exposed pregnancies). For major malformations, the summary estimate of adjusted ORs was attenuated in both the original (1.05, 95% CI .84–1.31, I^2^ 0%) and updated analyses (1.06, 95% CI .86–1.31, I^2^ 0%). The cohort study by Bateman *et al.*^4^ had the largest weight (78% in the adjusted analyses) followed by our study (11% in the adjusted analyses) (*Figure 4*). For heart malformations, the summary estimate of adjusted ORs was also attenuated in both the original (1.24, 95% CI .93–1.66, I^2^ 0%) and updated analyses (1.24, 95% CI .94–1.64, *I*^2^ 0%). Again, the cohort study by Bateman *et al.*^4^ had the largest weight (85% in adjusted analyses) followed by our study (10% in adjusted analyses) (see Supplementary data online, *Figure S14*).

## Discussion

In the present nationwide registry study of 805 368 pregnancies covering all pregnancies in Norway from 2005 to 2018, statin prescription fills in the first trimester were not associated with congenital malformations (Structured Graphical Abstract). Our results add to the growing body of evidence suggesting that statins and other LMAs are unlikely to be major teratogens.

### Interpretation of main results

Statin use in pregnancy has been linked to various adverse pregnancy outcomes, but the available human evidence has largely been inconclusive. Except for one large cohort study,^4^ the literature is dominated by case reports and case series with few exposed pregnancies, together with small-scale cohorts and case–control studies.^11,12,21^ In their systematic review and meta-analysis of five cohorts^4–9^ and one case–control study,^10^ Karadas *et al.*^11^ concluded that there was no evidence of a significant increase in the risk of major congenital malformations or heart defects after first-trimester statin exposure, when combining ORs adjusted for potential confounders. Our results align well with these findings, as we found that the odds of congenital malformations were not increased in models adjusted for age, parity, pre-pregnancy folate use, smoking in early pregnancy, the comorbidity index, and the comorbidity severity (co-medication) index.

Confounding by indication is a major problem in drug safety analyses.^22^ Women of fertile age using statins or other LMAs likely have an estimated high risk of ASCVD, possibly because of FH or other dyslipidaemias, diabetes mellitus, hypertension, or other pre-existing conditions, which may differ between study populations. Maternal diabetes mellitus, dyslipidaemia, or related conditions may increase the risk for congenital malformations independent of medication use.^23,24^ Selecting confounding factors to include in statistical models is therefore complicated.^22^ In the present work, we included analyses restricted to statin users with statin-discontinuer pregnancies as the reference group, which we believe accounts for confounding by indication to a larger degree than traditional analyses with non-exposed pregnancies as the reference group. Our results support this argument, as results with discontinuer pregnancies as the reference group were similar in the crude and adjusted analyses. It is remarkable that whilst the use of drug-discontinued pregnancies as a reference group is common for other drugs and outcomes, this approach is rarely employed when statins are analysed.^25,26^

Because statins are considered contraindicated in pregnancy, a single injection of the siRNA-based, liver-specific PCSK9 inhibitor inclisiran has been suggested as an option for cholesterol lowering during periods of pregnancy and breastfeeding.^27^ The mean half-life of inclisiran is ∼7 h, and the drug is cleared from circulation within 12–24 h, but its LDL cholesterol-lowering effect lasts 6–12 months.^28^ A single-course, *in vivo* base editing of the *PCSK9* gene in liver cells is also in development and may be a potential future strategy to treat FH.^29^ However, a recent Mendelian randomization study found that genetic, whole-body *PCSK9* loss-of-function mimicking PCSK9 inhibition-based therapy was associated with a higher risk of birth defects.^30^ In contrast, a recent disproportionality analysis in VigiBase, the WHO global pharmacovigilance database, found no signals of increased reporting of spontaneous abortion with alirocumab and evolocumab compared with the full database and statins.^31^ In that study, patterns of birth defects were not reported. Regardless, caution is still warranted for the use of PCSK9 inhibitors during pregnancy due to lack of formal studies in humans.

### Implications, importance, and generalizability

Our results have implications for market authorization holders and regulatory agencies such as the FDA and the European Medicines Agency (EMA), as they are responsible for the pregnancy and breastfeeding sections of drug labels, as well as for designing risk management plans aimed at addressing knowledge gaps regarding drug safety during pregnancy and breastfeeding. Notably, EMA continues to uphold stringent regulations on statin use during pregnancy through product information summary of product characteristics, package leaflet, and labelling.^32^

Our results also have implications for women of fertile age who are eligible for statins and healthcare providers, as they can assist in shared decision-making for drug therapy around the time of pregnancy and breastfeeding. For example, our study offers reassurance for women unknowingly exposed to statins during pregnancy; it is likely that the underlying maternal illness is more harmful for the developing fetus than the drug exposure. Moreover, for women with a very high risk of ASCVD, it is becoming increasingly clear that the benefits of treating the mother outweigh the potential risks for the offspring. For example, compared to men, young women with FH have a higher LDL cholesterol burden,^33^ and as many as 8% of women of fertile age (28–52 years) have experienced CVD events, half of which occurred around the time of pregnancy.^34^ Registry studies have also shown that the excess risk of CVD morbidity and mortality is especially pronounced among young women with FH,^35,36^ adding to the concern that we may have underestimated the consequences of the massive LDL cholesterol burden in early life and around the time of pregnancy.^34^

Considering the increasing use of statins and other LMAs among women of fertile age, continued monitoring of drug utilization and safety is important.^13^ Additionally, although a final conclusion has not yet been reached on statin’s effects to prevent pre-eclampsia, preliminary data from randomized controlled trials among women with high risk of pre-eclampsia suggest they are not associated with adverse maternal and neonatal outcomes when used in later trimesters of pregnancy.^37^

Overall, our data support that EMA and other regulatory bodies should reconsider the strongest warning against statin use during pregnancy, in alignment with recent FDA recommendations.^3^ Additionally, off-treatment periods around pregnancy should be kept as short as possible.

### Strengths and limitations

The present study has several strengths that are important to consider. First, by leveraging the national registries in Norway, we have coverage for all pregnancies in Norway in the study period, all prescriptions dispensed from Norwegian pharmacies, and most relevant comorbidities registered in two patient registries, ensuring that our findings are representative of the entire Norwegian population. Second, the extensive dataset provides a robust sample size that enhances the validity of our results, only surpassed by Bateman *et al.*^4^ Third, this study includes three distinct exposure groups, including discontinuers, allowing for a more nuanced analysis of the effects of statin use during pregnancy. Notably, the analyses using discontinuers as the reference group were more likely to be free from residual confounding, compared to conventional non-exposed analyses. Finally, the rates of congenital malformations observed in our study are consistent with Bateman *et al.*^4^ which enhances the credibility of our results.

We also acknowledge that there are several limitations of the present study. First, the study’s observational design limits the ability to establish causal relationships, and we cannot exclude the possibility of residual confounding, especially in the analyses with the non-exposed pregnancies as the reference group. Second, the findings may not be generalizable to non-Norwegian populations due to potential differences in healthcare systems, prescribing practices, and population demographics. Third, although the MBRN registers pregnancy-related information for all pregnancies ending after gestational week 12, we do not have data on spontaneous abortions occurring before this period. Furthermore, the registry data may be inaccurate or incomplete for certain variables. Nevertheless, information bias is probably low since the data collection was part of routine health care registrations; however, there was evidence of missing data for smoking status being related to both statin prescription fills and congenital malformations. Additionally, the low frequency of prescription fills during the first trimester of pregnancy and low number of congenital malformations may restrict statistical power. Our study therefore lacks well-powered granular data on associations between statin types, doses, and duration of therapy, and subgroups of congenital malformations. This limitation is common in comparable analyses,^11,12,21^ and for better-powered drug safety analyses, multinational registry studies should be conducted.

Finally, exposure misclassification cannot be excluded: the exposure is ‘prescription fill’, not actual drug use, but these are assumed to correlate reasonably well.^38^ Note, however, that information recorded in NorPD is based on drugs dispensed from pharmacies to patients, which is more indicative of drug use than databases that include all drugs prescribed by physicians.^39^ Despite the potential for misclassification, sensitivity analyses using different definitions of the exposure variable showed similar results to the main analyses, indicating the main exposure variable is likely robust.

### Conclusions

In summary, statin prescription fills in the first trimester of pregnancy was not associated with congenital malformations. However, low power may have limited our ability to detect weak but clinically meaningful associations. Therefore, our findings do not support a strong and independent relationship but do not exclude the possibility of notable risks or benefits.

## Supplementary Material

ehaf592_Supplementary_Data

## Declarations

### Data Availability

All relevant data are within the paper and its supporting information files. The authors may not share the study data due to regulations which restrict access and distribution to those with ethical and legal permission to use the data. The study material is available to other researchers upon an application to the relevant register holders.

### Pre-registered Clinical Trial Number

None supplied.
