# Supplementary material for: Statin use in pregnancy and risk of congenital malformations: a Norwegian nationwide study
Source: Eur Heart J. 2025 Aug 21;47(3):318–27. doi: 10.1093/eurheartj/ehaf592 (PMC12807567; doi:10.1093/eurheartj/ehaf592)
Supplement: ehaf592_Supplementary_Data [file ehaf592_supplementary_data.docx]

ONLINE SUPPLEMENTARY MATERIAL

**Statin use in pregnancy and risk of congenital malformations: a Norwegian nationwide study**

Jacob J. Christensen^1^, Kirsten B. Holven^1,2^, Martin P. Bogsrud^3^, Kjetil Retterstøl^1,4^, Jeanine E. Roeters van Lennep^5^, Trond M. Michelsen^6,7^, Marit B. Veierød^8^, Hedvig Nordeng^9,10^

1 Department of Nutrition, Institute of Basic Medical Sciences, University of Oslo, Oslo, Norway

2 Norwegian National Advisory Unit on Familial Hypercholesterolemia, Oslo University Hospital, Oslo, Norway

3 Unit for Cardiac and Cardiovascular Genetics, Oslo University Hospital, Oslo, Norway

4 The Lipid Clinic, Oslo University Hospital, Oslo, Norway

5 Department of Internal Medicine, Erasmus MC Cardiovascular Institute, Erasmus MC University Medical Center, Rotterdam, the Netherlands

6 Department of Obstetrics, Division of Obstetrics and Gynecology, Oslo University Hospital, Oslo, Norway

7 Faculty of Medicine, University of Oslo, Oslo, Norway

8 Oslo Centre for Biostatistics and Epidemiology, Department of Biostatistics, Institute of Basic Medical Sciences, University of Oslo, Oslo, Norway

9 PharmacoEpidemiology and Drug Safety Research Group, University of Oslo, Oslo, Norway

10 Department of Child Health and Development, Norwegian Institute of Public Health, Oslo, Norway

**Correspondence**: Jacob J. Christensen, PhD, e-mail: [j.j.christensen@medisin.uio.no](mailto:j.j.christensen@medisin.uio.no)

# Supplementary Methods

## Data measurements and variables

### Exposures

*Any LMA* was defined as ATC codes C10*, which includes C10A* [monotherapy] and C10B* [combination therapy] (Table S1).

*Statin LMAs* was defined as ATC codes C10AA* [statin monotherapy] and C10B* [statin combination therapy] (Table S1).

*Non-statin LMAs* was defined as ATC codes C10AB*, C10AC*, C10AD*, C10AX*, and C10BA*. Isolated use of non-statin LMAs had low prevalence in the present study population and was not used in any analyses (1).

Note that asterisk indicates inclusion of all subgroups within an ATC code (Table S1). For example, C10AA* includes all subcategories of C10AA, which corresponds to C10AA01 (Simvastatin), C10AA02 (Lovastatin), C10AA03 (Pravastatin), C10AA04 (Fluvastatin), C10AA05 (Atorvastatin), C10AA06 (Cerivastatin), C10AA07 (Rosuvastatin), and C10AA08 (Pitavastatin).

As reported previously, the main types of statins used in this population were simvastatin and atorvastatin (1). In Table S6, we give an overview of the distribution of types and doses across the exposure groups, and in Table S7, we give an overview of the distribution of number of prescription fills (a measure of duration of therapy) across the exposure groups.

### Outcomes

For *Any congenital malformation*, ICD-10 codes were derived from the EUROCAT subgroups of congenital anomalies (Version 2014, Guide 1.4, Chapter 3.3) (2), and were all codes within the Q chapter (Congenital malformations, deformations and chromosomal abnormalities), D82.1, P35.0-P35.1, P37.1, and D1810 (reproduced in Table S2). Note that the Q chapter does not include inborn errors of metabolism, and that we excluded pregnancies with offspring with chromosomal abnormalities.

In Table S2, we listed the prevalence of subgroups of congenital malformations across the entire study population. The exposure group-specific prevalences are low and thus undisclosed due to data privacy regulations.

For *Minor malformations*, ICD-10 codes were all codes listed in the EUROCAT exclusion list for minor malformations (Version 2014, Guide 1.4, Chapter 3.2) (2) (reproduced in Table S3).

For *Major malformations*, ICD-10 codes were codes for *Any congenital malformation*, excluding isolated minor malformations, as per EUROCAT standard (Table S2) (2).

For *Heart malformations*, ICD-10 codes were derived from the EUROCAT subgroups of congenital anomalies (Version 2014, Guide 1.4, Chapter 3.3) (2) (reproduced in Table S2), and were codes Q20-Q26, but not conditions such as Q25.0 (patent ductus arteriosus), and Q25.6 (congenital stenosis of the pulmonary artery) if gestational age <37 weeks, as per EUROCAT standard.

### Covariates

From NPR and KUHR, we derived co-morbidities registered any time before conception, including pre-pregnancy diabetes mellitus (ICD-10 codes E10-E14, and ICPC-2 codes T89 and T90), pre-pregnancy hypertension (ICD-10 codes I10-I15, and ICPC-2 codes K85 and K86), and pre-pregnancy cardiovascular disease (CVD, ICD-10 codes in I excl. I10-I15, and ICPC-2 codes in K excl. K85 and K86). We also derived co-morbidity variables based on diagnoses 6-12 months before conceptions or during 1^st^ trimester; these were used in sensitivity analyses (data not shown).

From NorPD, we derived co-medication registered 6-12 months before conception, including pre-pregnancy use of diabetes drugs, defined as ATC codes starting with A10*; pre-pregnancy use of anti-thrombotic agents, defined as B01*; and pre-pregnancy use of CVD drugs, defined as C* excl. C10* (Table S4). We also derived co-medication variables based on co-medication in 1^st^ trimester; these were used in sensitivity analyses (data not shown).

# Supplementary References

1. Christensen JJ, Bogsrud MP, Holven KB, et al. Use of statins and other lipid-modifying agents across pregnancy: A nationwide drug utilization study in Norway in 2005–2018. Atherosclerosis. Elsevier; 2022;36825–34. https://doi.org/10.1016/j.atherosclerosis.2022.11.022.

2. European Platform on Rare Disease Registration. EUROCAT. 2025. https://eu-rd-platform.jrc.ec.europa.eu/eurocat/data-collection/guidelines-for-data-registration_en.

# Supplementary Tables

## Table S1

**Table S1**. ATC codes for defining the exposure variables.

| **Anatomical main group / therapeutic subgroup / pharmacological subgroup / chemical subgroup** | **Chemical substance** |
| --- | --- |
| C Cardiovascular System |  |
| C10 LMAs |  |
| C10A LMAs, plain |  |
| C10AA HMG CoA reductase inhibitors | C10AA01 Simvastatin |
| C10AA HMG CoA reductase inhibitors | C10AA02 Lovastatin |
| C10AA HMG CoA reductase inhibitors | C10AA03 Pravastatin |
| C10AA HMG CoA reductase inhibitors | C10AA04 Fluvastatin |
| C10AA HMG CoA reductase inhibitors | C10AA05 Atorvastatin |
| C10AA HMG CoA reductase inhibitors | C10AA06 Cerivastatin |
| C10AA HMG CoA reductase inhibitors | C10AA07 Rosuvastatin |
| C10AA HMG CoA reductase inhibitors | C10AA08 Pitavastatin |
| C10AB Fibrates | C10AB01 Clofibrate |
| C10AB Fibrates | C10AB02 Bezafibrate |
| C10AB Fibrates | C10AB03 Aluminium clofibrate |
| C10AB Fibrates | C10AB04 Gemfibrozil |
| C10AB Fibrates | C10AB05 Fenofibrate |
| C10AB Fibrates | C10AB06 Simfibrate |
| C10AB Fibrates | C10AB07 Ronifibrate |
| C10AB Fibrates | C10AB08 Ciprofibrate |
| C10AB Fibrates | C10AB09 Etofibrate |
| C10AB Fibrates | C10AB10 Clofibride |
| C10AB Fibrates | C10AB11 Choline fenofibrate |
| C10AC Bile acid sequestrants | C10AC01 Colestyramine |
| C10AC Bile acid sequestrants | C10AC02 Colestipol |
| C10AC Bile acid sequestrants | C10AC03 Colextran |
| C10AC Bile acid sequestrants | C10AC04 Colesevelam |
| C10AD Nicotinic acid and derivatives | C10AD01 Niceritrol |
| C10AD Nicotinic acid and derivatives | C10AD02 Nicotinic acid |
| C10AD Nicotinic acid and derivatives | C10AD03 Nicofuranose |
| C10AD Nicotinic acid and derivatives | C10AD04 Aluminium nicotinate |
| C10AD Nicotinic acid and derivatives | C10AD05 Nicotinyl alcohol (pyridylcarbinol) |
| C10AD Nicotinic acid and derivatives | C10AD06 Acipimox |
| C10AD Nicotinic acid and derivatives | C10AD52 Nicotinic acid, combinations |
| C10AX Other LMAs | C10AX01 Dextrothyroxine |
| C10AX Other LMAs | C10AX02 Probucol |
| C10AX Other LMAs | C10AX03 Tiadenol |
| C10AX Other LMAs | C10AX05 Meglutol |
| C10AX Other LMAs | C10AX06 Omega-3-triglycerides incl. other esters and acids |
| C10AX Other LMAs | C10AX07 Magnesium pyridoxal 5-phosphate glutamate |
| C10AX Other LMAs | C10AX08 Policosanol |
| C10AX Other LMAs | C10AX09 Ezetimibe |
| C10AX Other LMAs | C10AX10 Alipogene tiparvovec |
| C10AX Other LMAs | C10AX11 Mipomersen |
| C10AX Other LMAs | C10AX12 Lomitapide |
| C10AX Other LMAs | C10AX13 Evolocumab |
| C10AX Other LMAs | C10AX14 Alirocumab |
| C10AX Other LMAs | C10AX15 Bempedoic acid |
| C10AX Other LMAs | C10AX16 Inclisiran |
| C10B LMAs, combinations |  |
| C10BA HMG CoA reductase inhibitors in combination with other LMAs | C10BA01 Lovastatin and nicotinic acid |
| C10BA HMG CoA reductase inhibitors in combination with other LMAs | C10BA02 Simvastatin and ezetimibe |
| C10BA HMG CoA reductase inhibitors in combination with other LMAs | C10BA03 Pravastatin and fenofibrate |
| C10BA HMG CoA reductase inhibitors in combination with other LMAs | C10BA04 Simvastatin and fenofibrate |
| C10BA HMG CoA reductase inhibitors in combination with other LMAs | C10BA05 Atorvastatin and ezetimibe |
| C10BA HMG CoA reductase inhibitors in combination with other LMAs | C10BA06 Rosuvastatin and ezetimibe |
| C10BA HMG CoA reductase inhibitors in combination with other LMAs | C10BA07 Rosuvastatin and omega-3 fatty acids |
| C10BA HMG CoA reductase inhibitors in combination with other LMAs | C10BA08 Atorvastatin and omega-3 fatty acids |
| C10BA HMG CoA reductase inhibitors in combination with other LMAs | C10BA09 Rosuvastatin and fenofibrate |
| C10BX HMG CoA reductase inhibitors, other combinations | C10BX01 Simvastatin and acetylsalicylic acid |
| C10BX HMG CoA reductase inhibitors, other combinations | C10BX02 Pravastatin and acetylsalicylic acid |
| C10BX HMG CoA reductase inhibitors, other combinations | C10BX03 Atorvastatin and amlodipine |
| C10BX HMG CoA reductase inhibitors, other combinations | C10BX04 Simvastatin, acetylsalicylic acid and ramipril |
| C10BX HMG CoA reductase inhibitors, other combinations | C10BX05 Rosuvastatin and acetylsalicylic acid |
| C10BX HMG CoA reductase inhibitors, other combinations | C10BX06 Atorvastatin, acetylsalicylic acid and ramipril |
| C10BX HMG CoA reductase inhibitors, other combinations | C10BX07 Rosuvastatin, amlodipine and lisinopril |
| C10BX HMG CoA reductase inhibitors, other combinations | C10BX08 Atorvastatin and acetylsalicylic acid |
| C10BX HMG CoA reductase inhibitors, other combinations | C10BX09 Rosuvastatin and amlodipine |
| C10BX HMG CoA reductase inhibitors, other combinations | C10BX10 Rosuvastatin and valsartan |
| C10BX HMG CoA reductase inhibitors, other combinations | C10BX11 Atorvastatin, amlodipine and perindopril |
| C10BX HMG CoA reductase inhibitors, other combinations | C10BX12 Atorvastatin, acetylsalicylic acid and perindopril |
| C10BX HMG CoA reductase inhibitors, other combinations | C10BX13 Rosuvastatin, perindopril and indapamide |
| C10BX HMG CoA reductase inhibitors, other combinations | C10BX14 Rosuvastatin, amlodipine and perindopril |
| C10BX HMG CoA reductase inhibitors, other combinations | C10BX15 Atorvastatin and perindopril |
| C10BX HMG CoA reductase inhibitors, other combinations | C10BX16 Rosuvastatin and fimasartan |
| C10BX HMG CoA reductase inhibitors, other combinations | C10BX17 Rosuvastatin and ramipril |
| C10BX HMG CoA reductase inhibitors, other combinations | C10BX18 Atorvastatin, amlodipine and ramipril |

## Table S2

**Table S2**. EUROCAT subgroups of congenital malformations, after EUROCAT version 2014 (Guide 1.4, Chapter 3.3).

| **EUROCAT Subgroups** | **ICD-10 codes** | **Excluded minor anomalies** | **Overall prevalence of major malformations, N = 805,368** |
| --- | --- | --- | --- |
| ***All anomalies*** | Q-chapter, D215, D821, D1810, P350, P351, P371 | All minor anomalies (see separate table) | 19,376 (2.41%) |
| **Nervous system** | Q00, Q01, Q02, Q03, Q04, Q05, Q06, Q07 | Q0461, Q0782 | 1,394 (0.17%) |
| **Eye** | Q10, Q11, Q12, Q13, Q14, Q15 | Q101, Q102, Q103, Q105, Q135 | 167 (0.02%) |
| **Ear, face and neck** | Q16, Q17, Q18 | Q170, Q171, Q172, Q173, Q174, Q175, Q179, Q180, Q181, Q182, Q184, Q185, Q186, Q187, Q1880, Q189 | 191 (0.02%) |
| **Congenital Heart Defects** | Q20, Q21, Q22, Q23, Q24, Q25, Q26 | Q2111, Q250 if GA <37 weeks, Q2541, Q256 if GA<37 weeks, Q261 | 6,291 (0.78%) |
| **Respiratory** | Q300, Q32, Q33, Q34 | Q314, Q315, Q320, Q331 | 514 (0.06%) |
| **Oro-facial clefts** | Q35, Q36, Q37 |  | 1,384 (0.17%) |
| **Digestive system** | Q38, Q39, Q40, Q41, Q42, Q43, Q44, Q45, Q790 | Q381, Q382, Q3850, Q400, Q401, Q4021, Q430, Q4320, Q4381, Q4382 | 1,311 (0.16%) |
| **Abdominal wall defects** | Q792, Q793, Q795 |  | 500 (0.06%) |
| **Urinary** | Q60, Q61, Q62, Q63, Q64, Q794 | Q610, Q627, Q633 | 2,139 (0.27%) |
| **Genital** | Q50, Q51, Q52, Q54, Q55, Q56 | Q523, Q525, Q527, Q5520, Q5521 | 2,084 (0.26%) |
| **Limb** | Q65, Q66, Q67, Q68, Q69, Q70, Q71, Q72, Q73, Q74 | Q653, Q654, Q655, Q656, Q662, Q663, Q664, Q665, Q666, Q667, Q668, Q669, Q670, Q671, Q672, Q673, Q674, Q675, Q676, Q677, Q678, Q680, Q6810, Q6821, Q683, Q684, Q685, Q7400 | 4,562 (0.57%) |
| **Other anomalies / syndromes** | Q7402, Q77, Q7800, Q782, Q783, Q784, Q785, Q786, Q787, Q788, Q750, Q7980, Q893, Q894, Q80, Q81, Q82, Q8726, Q0435, Q411, Q412, Q418, Q710, Q712, Q713, Q720, Q722, Q723, Q730, Q793, Q795, Q7980, Q7982, Q8706, Q206, Q240, Q3381, Q890, Q893, Q86, P350, P351, P371, Q860, Q8680, P350, P351, P371, Q4471, Q6190, Q7484, Q751, Q754, Q7581, Q87, Q936, D821 |  | 1,534 (0.19%) |

## Table S3

**Table S3**. Minor anomalies for exclusion, after EUROCAT version 2014 (Guide 1.4, Chapter 3.2) (2).

| **Diagnosis** | **ICD-10 codes** |
| --- | --- |
| Compression facies | Q671 |
| Depressions in skull | Q6740 |
| Dolichocephaly | Q672 |
| Dysmorphic face | Q189 |
| Facial asymmetry | Q670 |
| Plagiocephaly — head asymmetry | Q673 |
| Macrocephalus | Q753 |
| Other congenital deformities of skull, face and jaw | Q674 |
| Blue sclera | Q135 |
| Congenital ectropion | Q101 |
| Congenital entropion | Q102 |
| Crocodile tears | Q0782 |
| Hypertelorism | Q752 |
| Other congenital malformations of eyelid | Q103 |
| Stenosis or stricture of lacrimal duct | Q105 |
| Synophrys | Q1880 |
| Accesorry auricle, preauricular appendage, tag or lobule | Q170 |
| Asymmetric size | Q173 |
| Bat ear, prominent ear | Q175 |
| Double lobule | Q170 |
| Lack of helical fold | Q173 |
| Low set ears | Q174 |
| Macrotia | Q171 |
| Microtia | Q172 |
| Posterior angulation | Q173 |
| Preauricular sinus or cyst | Q181 |
| Primitive shape | Q173 |
| Protuberant ears | Q173 |
| Unspecified and minor malformation of ear | Q179 |
| Deviation of nasal septum | Q6741 |
| Dysmorphic nose | Q189 |
| High arched palate | Q3850 |
| Macrocheilia | Q186 |
| Macroglossia | Q382 |
| Macrostomia | Q184 |
| Microcheilia | Q187 |
| Microstomia | Q185 |
| Retrognathia | Q674 |
| Tongue tie or cyst of tongue | Q381 |
| Congenital malformation of face and neck, unspecified | Q189 |
| Other branchial cleft malformations | Q182 |
| Preauricular sinus or cyst | Q181 |
| Sinus, fistula or cyst of branchial cleft | Q180 |
| Torticollis | Q680 |
| Accessorry carpal bones | Q7400 |
| Clinodactyly (5th finger) | Q6810 |
| Enlarged or hypertrophic nails | Q845 |
| Single/abnormal palmar crease | Q8280 |
| Clicking hip, subluxation or unstable hip | Q653 |
| Clicking hip, subluxation or unstable hip | Q654 |
| Clicking hip, subluxation or unstable hip | Q655 |
| Clicking hip, subluxation or unstable hip | Q656 |
| Clubfoot of postural origin - other cong deformities of feet | Q668 |
| Congenital deformity of feet, unspecified | Q669 |
| Congenital pes planus | Q665 |
| Enlarged or hypertrophic nails | Q845 |
| Hallux varus — other congenital varus deformities of feet | Q663 |
| Metatarsus varus — other congenital valgus deformities of feet | Q666 |
| Metatarsus varus or metatarsus adductus | Q662 |
| Pes cavus | Q667 |
| Talipes or pes calcaneovalgus | Q664 |
| Accessory nipples | Q833 |
| Mongoloid spot (whites) | Q8252 |
| Neavus flammeus | Q8250 |
| Pigmented naevus — congenital non-neoplastic naevus | Q825 |
| Strawberry naevus | Q8251 |
| Absence of rib | Q7660 |
| Accessory rib | Q7662 |
| Cervical rib | Q765 |
| Congenital bowing of femur | Q683 |
| Congenital bowing of fibula and tibia | Q684 |
| Congenital bowing of long bones of leg, unspecified | Q685 |
| Congenital deformity of spine | Q675 |
| Congenital lordosis, postural | Q7643 |
| Depressed sternum | Q676 |
| Genu recurvatum | Q6821 |
| Prominent sternum | Q677 |
| Shieldlike chest, other congenital deformities of chest | Q678 |
| Spina bifida occulta | Q760 |
| Sternum bifidum | Q7671 |
| Single congenital cerebral cyst | Q0461 |
| Absence or hypoplasia of umbilical artery, single umbilical artery | Q270 |
| Patent ductus arteriosus if GA < 37 weeks | Q250 |
| Patent or persistent foramen ovale | Q2111 |
| Peripheral pulmonary artery stenosis if GA < 37 weeks | Q256 |
| Persistent left superior vena cava | Q261 |
| Persistent right aortic arch | Q2541 |
| Accessory lobe of lung | Q331 |
| Azygos lobe of lung | Q3310 |
| Congenital laryngeal stridor | Q314 |
| Laryngomalacia | Q314 |
| Laryngomalacia | Q315 |
| Tracheomalacia | Q320 |
| Functional gastro-intestinal disorders | Q4021 |
| Functional gastro-intestinal disorders | Q4320 |
| Functional gastro-intestinal disorders | Q4381 |
| Functional gastro-intestinal disorders | Q4382 |
| Hiatus hernia | Q401 |
| Meckel's diverticulum | Q430 |
| Pyloric stenosis | Q400 |
| Hyperplastic and giant kidney | Q633 |
| Single renal cyst | Q610 |
| Vesico-ureteral-renal reflux | Q627 |
| Bifid scrotum | Q5521 |
| Congenital malformation of vulva | Q527 |
| Fusion of labia | Q525 |
| Hymen imperforatum | Q523 |
| Retractile testis | Q5520 |
| Undescended testicle | Q53 |
| Congenital malformation, unspecified | Q899 |
| Balanced translocations or inversions in normal individuals | Q950 |
| Balanced translocations or inversions in normal individuals | Q951 |

## Table S4

**Table S4**. ATC codes for defining the co-medication variables.

| **Anatomical main group / therapeutic subgroup** | **Pharmacological subgroup** |
| --- | --- |
| A Alimentary Tract And Metabolism |  |
| A10 drugs used in diabetes | A10A insulins and analogues |
| A10 drugs used in diabetes | A10B blood glucose lowering drugs, excl. insulins |
| B Blood And Blood Forming Organs |  |
| B01 antithrombotic agents | B01A antithrombotic agents |
| C Cardiovascular System |  |
| C02 antihypertensives | C02A Antiadrenergic agents, centrally acting |
| C02 antihypertensives | C02B Antiadrenergic agents, ganglion-blocking |
| C02 antihypertensives | C02C Antiadrenergic agents, peripherally acting |
| C02 antihypertensives | C02D Arteriolar smooth muscle, agents acting on |
| C02 antihypertensives | C02K Other antihypertensives |
| C02 antihypertensives | C02L Antihypertensives and diuretics in combination |
| C02 antihypertensives | C02N Combinations of antihypertensives in ATC gr. C02 |
| C03 diuretics | C03A low-ceiling diuretics, thiazides |
| C03 diuretics | C03B low-ceiling diuretics, excl. thiazides |
| C03 diuretics | C03C high-ceiling diuretics |
| C03 diuretics | C03D potassium-sparing agents |
| C03 diuretics | C03E diuretics and potassium-sparing agents in combination |
| C03 diuretics | C03X other diuretics |
| C04 peripheral vasodilators | C04A peripheral vasodilators |
| C07 beta blocking agents | C07A beta blocking agents |
| C07 beta blocking agents | C07B beta blocking agents and thiazides |
| C07 beta blocking agents | C07C beta blocking agents and other diuretics |
| C07 beta blocking agents | C07D beta blocking agents, thiazides and other diuretics |
| C07 beta blocking agents | C07E beta blocking agents and vasodilators |
| C07 beta blocking agents | C07F beta blocking agents, other combinations |
| C08 calcium channel blockers | C08C selective calcium channel blockers with mainly vascular effects |
| C08 calcium channel blockers | C08D selective calcium channel blockers with direct cardiac effects |
| C08 calcium channel blockers | C08E non-selective calcium channel blockers |
| C08 calcium channel blockers | C08G calcium channel blockers and diuretics |
| C09 agents acting on the renin-angiotensin system | C09A ACE inhibitors, plain |
| C09 agents acting on the renin-angiotensin system | C09B ACE inhibitors, combinations |
| C09 agents acting on the renin-angiotensin system | C09C angiotensin II receptor blockers (ARBs), plain |
| C09 agents acting on the renin-angiotensin system | C09D angiotensin II receptor blockers (ARBs), combinations |
| C09 agents acting on the renin-angiotensin system | C09X other agents acting on the renin-angiotensin system |

## Table S5

**Table S5**. Characteristics of pregnancies exposed or not exposed to statins in 1^st^ trimester: focus on indications for therapy, co-medication and co-morbidities.

|  | **Non-exposed pregnancies, N = 803,830** | **Discontinuer pregnancies, N = 1,255** | **Exposed pregnancies, N = 283** |
| --- | --- | --- | --- |
| *Indication for prescription fill^1^* | | | |
| Established ASCVD^2^ | 236 (<0.1) | 288 (23) | 82 (29) |
| High risk of ASCVD^3^ | 705 (<0.1) | 850 (68) | 183 (65) |
| FH or other dyslipidemia^4^ | 32 (<0.1) | 103 (8.2) | 18 (6.4) |
| *Co-medication^5^* | | | |
| Co-morbidity severity index | | | |
| 0 | 779,235 (97) | 923 (74) | 180 (64) |
| 1 | 23,689 (2.9) | 247 (20) | 70 (25) |
| ≥ 2 | 906 (0.1) | 85 (6.8) | 33 (12) |
| Diabetes drugs^6^ | | | |
| 6-12 mo before conception | 5,852 (0.7) | 144 (11) | 45 (16) |
| 1st trimester of pregnancy | 5,590 (0.7) | 133 (11) | 51 (18) |
| Anti-thrombotic agents^7^ | | | |
| 6-12 mo before conception | 4,714 (0.6) | 107 (8.5) | 30 (11) |
| 1st trimester of pregnancy | 14,330 (1.8) | 99 (7.9) | 46 (16) |
| Cardiovascular system incl. hypertension^8^ | | | |
| 6-12 mo before conception | 14,957 (1.9) | 171 (14) | 64 (23) |
| 1st trimester of pregnancy | 5,811 (0.7) | 91 (7.3) | 68 (24) |
| Anti-inflammatory agents^9^ | | | |
| 6-12 mo before conception | 64,177 (8.0) | 190 (15) | 37 (13) |
| 1st trimester of pregnancy | 13,291 (1.7) | 45 (3.6) | 16 (5.7) |
| Analgesics^10^ | | | |
| 6-12 mo before conception | 55,086 (6.9) | 195 (16) | 44 (16) |
| 1st trimester of pregnancy | 20,637 (2.6) | 74 (5.9) | 31 (11) |
| Psycholeptics^11^ | | | |
| 6-12 mo before conception | 23,162 (2.9) | 100 (8.0) | 24 (8.5) |
| 1st trimester of pregnancy | 12,504 (1.6) | 47 (3.7) | 15 (5.3) |
| Psychoanaleptics^12^ | | | |
| 6-12 mo before conception | 22,674 (2.8) | 92 (7.3) | 36 (13) |
| 1st trimester of pregnancy | 10,682 (1.3) | 32 (2.5) | 24 (8.5) |
| *Co-morbidities^13^* | | | |
| Co-morbidity index | | | |
| 0 | 767,312 (95) | 896 (71) | 169 (60) |
| 1 | 34,809 (4.3) | 294 (23) | 79 (28) |
| ≥ 2 | 1,709 (0.2) | 65 (5.2) | 35 (12) |
| Diabetes mellitus^14^ | | | |
| Anytime before conception | 5,775 (0.7) | 162 (13) | 51 (18) |
| 6-12 mo before conception | 3,248 (0.4) | 123 (9.8) | 40 (14) |
| 1st trimester of pregnancy | 3,384 (0.4) | 119 (9.5) | 47 (17) |
| Hyperlipidemia^15^ | | | |
| Anytime before conception | 4,344 (0.5) | 778 (62) | 136 (48) |
| 6-12 mo before conception | 647 (<0.1) | 328 (26) | 46 (16) |
| 1st trimester of pregnancy | 267 (<0.1) | 118 (9.4) | 39 (14) |
| Other endocrine, metabolic and nutritional diagnoses^16^ | | | |
| Anytime before conception | 50,699 (6.3) | 235 (19) | 60 (21) |
| 6-12 mo before conception | 14,789 (1.8) | 83 (6.6) | 16 (5.7) |
| 1st trimester of pregnancy | 13,306 (1.7) | 58 (4.6) | 21 (7.4) |
| Hypertension^17^ | | | |
| Anytime before conception | 11,357 (1.4) | 117 (9.3) | 47 (17) |
| 6-12 mo before conception | 2,704 (0.3) | 42 (3.3) | 16 (5.7) |
| 1st trimester of pregnancy | 2,151 (0.3) | 42 (3.3) | 20 (7.1) |
| Other cardiovascular diagnoses^18^ | | | |
| Anytime before conception | 21,146 (2.6) | 147 (12) | 55 (19) |
| 6-12 mo before conception | 3,010 (0.4) | 55 (4.4) | 21 (7.4) |
| 1st trimester of pregnancy | 2,652 (0.3) | 36 (2.9) | 27 (9.5) |
| n (%) for all variables. Abbreviations: ATC, the WHO’s Anatomical Therapeutic Chemical Classification System; ICD/ICD-10, the International Classification of Diseases, version 10; ICPC/ICPC-2, the International Classification of Primary Care-2; KUHR, the Norway Control and Payment of Health Reimbursement Database; NorPD, the Norwegian Prescription Registry; NPR, the Norwegian Patient Registry. | | | |
| ^1^Reimbursement codes as registered in NorPD from start of the registry to the end of first trimester | | | |
| ^2^Secondary prevention; reimbursement code -26 in ICD/ICPC | | | |
| ^3^Primary prevention; reimbursement code -27 in ICD/ICPC | | | |
| ^4^Reimbursement codes E78 in ICD, or T93 in ICPC | | | |
| ^5^Derived from NorPD | | | |
| ^6^Defined as ATC codes starting with A10* | | | |
| ^7^Defined as ATC codes starting with B01* | | | |
| ^8^Defined as ATC codes starting with C* excl. C10* | | | |
| ^9^Defined as ATC codes starting with M01* | | | |
| ^10^Defined as ATC codes starting with N02* | | | |
| ^11^Defined as ATC codes starting with N05* | | | |
| ^12^Defined as ATC codes starting with N06* | | | |
| ^13^Derived from NPR and KUHR | | | |
| ^14^Defined as ICD-10 codes E10*-E14*, and ICPC-2 codes T89* and T90* | | | |
| ^15^Defined as ICPC-2 code T93* | | | |
| ^16^Defined as all ICPC-2 codes in T* excl. T89*, T90* and T93* | | | |
| ^17^Defined as ICD-10 codes I10*-I15*, and ICPC-2 codes K85* and K86* | | | |
| ^18^Defined as ICD-10 codes in I* excl. I10*-I15*, and ICPC-2 codes in K* excl. K85* and K86* | | | |

## Table S6

**Table S6**. Distribution of pregnancies exposed to prescription fills of different LMA types and doses (monotherapy only, C10A group) registered from start of NorPD to the end of first trimester, by exposure groups (exposed to statins in 1^st^ trimester).

|  | **Non-exposed pregnancies, N = 803,830** | **Discontinuer pregnancies, N = 1,255** | **Exposed pregnancies, N = 283** |
| --- | --- | --- | --- |
| Simvastatin | 883 (0.1) | 846 (67) | 174 (61) |
| Simvastatin 10mg | 181 (<0.1) | 128 (10) | 31 (11) |
| Simvastatin 20mg | 489 (<0.1) | 477 (38) | 101 (36) |
| Simvastatin 40mg | 328 (<0.1) | 421 (34) | 77 (27) |
| Simvastatin 80mg | 30 (<0.1) | 72 (5.7) | 11 (3.9) |
| Atorvastatin | 455 (<0.1) | 655 (52) | 166 (59) |
| Atorvastatin 10mg | 153 (<0.1) | 201 (16) | 46 (16) |
| Atorvastatin 20mg | 169 (<0.1) | 298 (24) | 66 (23) |
| Atorvastatin 40mg | 174 (<0.1) | 316 (25) | 76 (27) |
| Atorvastatin 80mg | 62 (<0.1) | 110 (8.8) | 32 (11) |
| Rosuvastatin | 29 (<0.1) | 105 (8.4) | 17 (6.0) |
| Rosuvastatin 5mg | 10 (<0.1) | 28 (2.2) |  |
| Rosuvastatin 10mg | 11 (<0.1) | 40 (3.2) | 8 (2.8) |
| Rosuvastatin 20mg | 9 (<0.1) | 47 (3.7) | 6 (2.1) |
| Rosuvastatin 40mg | 7 (<0.1) | 33 (2.6) |  |
| Other statin LMAs | 78 (<0.1) | 85 (6.8) | 24 (8.5) |
| Other statin LMAs 20mg | 30 (<0.1) | 45 (3.6) | 15 (5.3) |
| Other statin LMAs 40mg | 31 (<0.1) | 32 (2.5) | 10 (3.5) |
| Other statin LMAs 80mg | 27 (<0.1) | 25 (2.0) | 6 (2.1) |
| Ezetimibe 10 mg | 61 (<0.1) | 142 (11) | 19 (6.7) |
| Colestyramine 4 g | 371 (<0.1) | 19 (1.5) |  |
| Omega-3^1^ | 93 (<0.1) | 8 (0.6) |  |
| Other non-statin LMAs^1^ | 27 (<0.1) | 16 (1.3) |  |
| n (%) for all variables. Abbreviations: LMA, lipid-modifying agents; NorPD, the Norwegian Prescription Registry. | | | |
| ^1^For omega-3, doses are unspecified. For other non-statin LMAs, only aggregated data are shown due to large variation in doses and administration routes. | | | |

## Table S7

**Table S7**. Distribution of per pregnancy number of prescription fills (a measure of duration of therapy) of different LMA types and doses (monotherapy only, C10A group) registered from start of NorPD to the end of first trimester, by exposure groups (exposed to statins in 1^st^ trimester).

|  | **Non-exposed pregnancies, N = 803,830** | **Discontinuer pregnancies, N = 1,255** | **Exposed pregnancies, N = 283** |
| --- | --- | --- | --- |
| Simvastatin | 2 (1-5) | 4 (2-9) | 6 (3-11) |
| Simvastatin 10mg | 1 (1-4) | 2 (1-7) | 7 (4-11) |
| Simvastatin 20mg | 2 (1-4) | 3 (1-7) | 4 (2-11) |
| Simvastatin 40mg | 2 (1-7) | 6 (2-11) | 6 (4-13) |
| Simvastatin 80mg | 3 (2-7) | 12 (7-16) | 8 (7-16) |
| Atorvastatin | 3 (1-7) | 5 (2-12) | 6 (3-12) |
| Atorvastatin 10mg | 2 (1-4) | 3 (1-6) | 4 (3-8) |
| Atorvastatin 20mg | 2 (1-5) | 4 (2-10) | 4 (3-9) |
| Atorvastatin 40mg | 4 (2-9) | 8 (3-14) | 8 (4-16) |
| Atorvastatin 80mg | 4 (2-10) | 8 (4-18) | 9 (6-12) |
| Rosuvastatin | 9 (4-21) | 13 (7-27) | 11 (8-17) |
| Rosuvastatin 5mg | 7 (5-14) | 11 (10-15) | 13 (10-22) |
| Rosuvastatin 10mg | 6 (1-9) | 14 (9-27) | 10 (6-14) |
| Rosuvastatin 20mg | 19 (9-19) | 10 (3-26) | 11 (8-20) |
| Rosuvastatin 40mg | 22 (22-29) | 22 (9-30) | 10 (10-10) |
| Other statin LMAs | 5 (2-10) | 5 (2-13) | 12 (6-22) |
| Other statin LMAs 20mg | 2 (2-6) | 5 (2-14) | 7 (1-17) |
| Other statin LMAs 40mg | 4 (3-8) | 5 (1-9) | 10 (7-14) |
| Other statin LMAs 80mg | 10 (6-26) | 5 (3-9) | 20 (13-26) |
| Ezetimibe 10 mg | 11 (6-18) | 22 (13-33) | 24 (16-48) |
| Colestyramine 4 g | 1 (1-2) | 5 (4-14) |  |
| Omega-3^1^ | 1 (1-4) | 22 (8-35) | 18 (13-34) |
| Other non-statin LMAs^1^ | 2 (1-10) | 36 (31-41) | 58 (30-85) |
| Median (25-75 percentile) for all variables. Abbreviations: LMA, lipid-modifying agents; NorPD, the Norwegian Prescription Registry. | | | |
| ^1^For omega-3, doses are unspecified. For other non-statin LMAs, only aggregated data are shown due to large variation in doses and administration routes. | | | |

# Supplementary Figures

## Figure S1

**
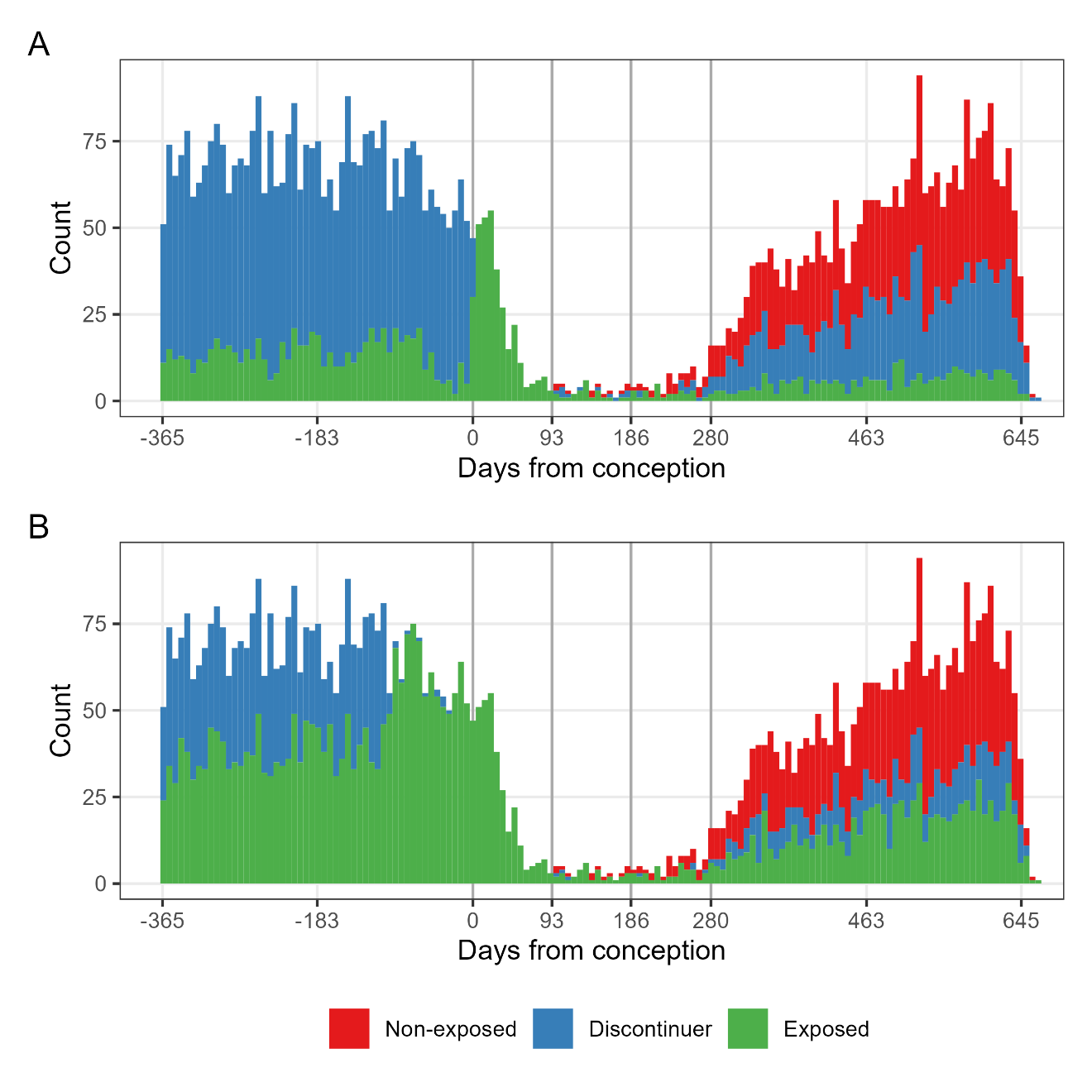
**

**Figure S1**. *Prescription fills for statins one year before conception, during pregnancy, and one year after giving birth.* The figure shows the number of prescriptions fills for statins across pregnancy in 2-week bins, where zero on the x-axis denote estimated time of conception. Fill color represents the three study groups, as described in the legend. In panel A (the main exposure of interest), the exposed group was defined as prescription fill in first trimester (between conception and 93 days into pregnancy); discontinuers were defined as prescription fills up to one year before conception, but not during first trimester; and non-exposed was defined as no prescription fills during first trimester and up to one year before conception. Panel B shows the same for the first trimester carryover exposure, which was defined as prescription fill package durations estimated to carry over into early pregnancy.

## Figure S2


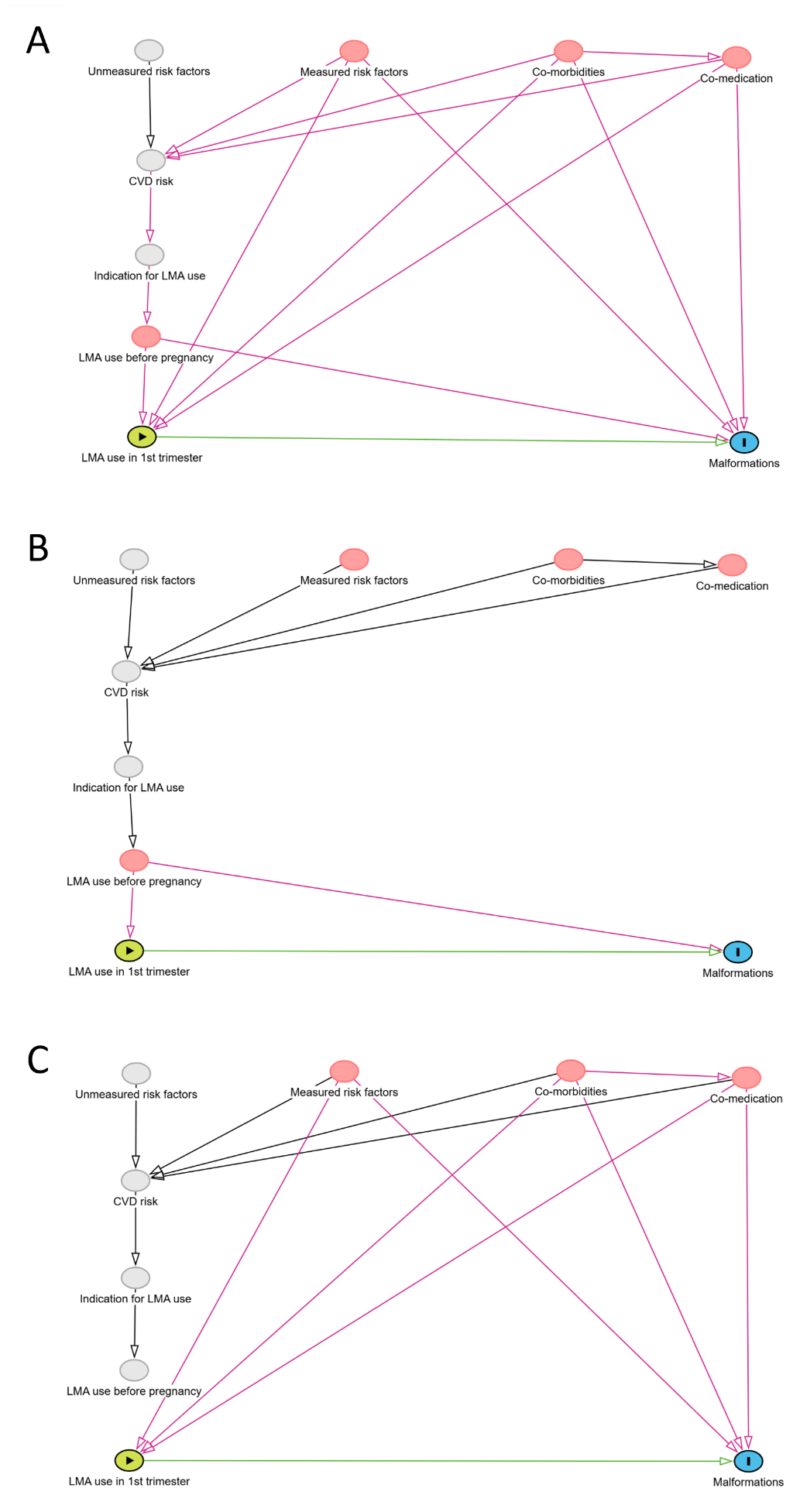


**Figure S2**. *Directed acyclic graphs for analysis of the association between 1^st^ trimester LMAs use and congenital malformations*. For the model in panel A, the minimal sufficient adjustment set for estimating the total effect of 1^st^ trimester LMA use on malformations was co-morbidities incl. diabetes, hypertension, and CVD; co-medication incl. medications used to treat such co-morbidities; measured risk factors such as age and parity; and LMA use before pregnancy. This model corresponded to the adjusted analysis with discontinuers as the reference group. For the model in panel B, the minimal sufficient adjustment set was LMA use before pregnancy. This model corresponded to the crude analysis with discontinuers as the reference group. For the model in panel C, the minimal sufficient adjustment set was co-morbidities, co-medication, and measured risk factors. This model corresponded to the adjusted analysis with the non-exposed population as reference group. We used dagitty.net to create the graphs and identify the minimal sufficient adjustment sets. Abbreviations: CVD, cardiovascular disease; LMA, lipid-modifying agents.

## Figure S3

**
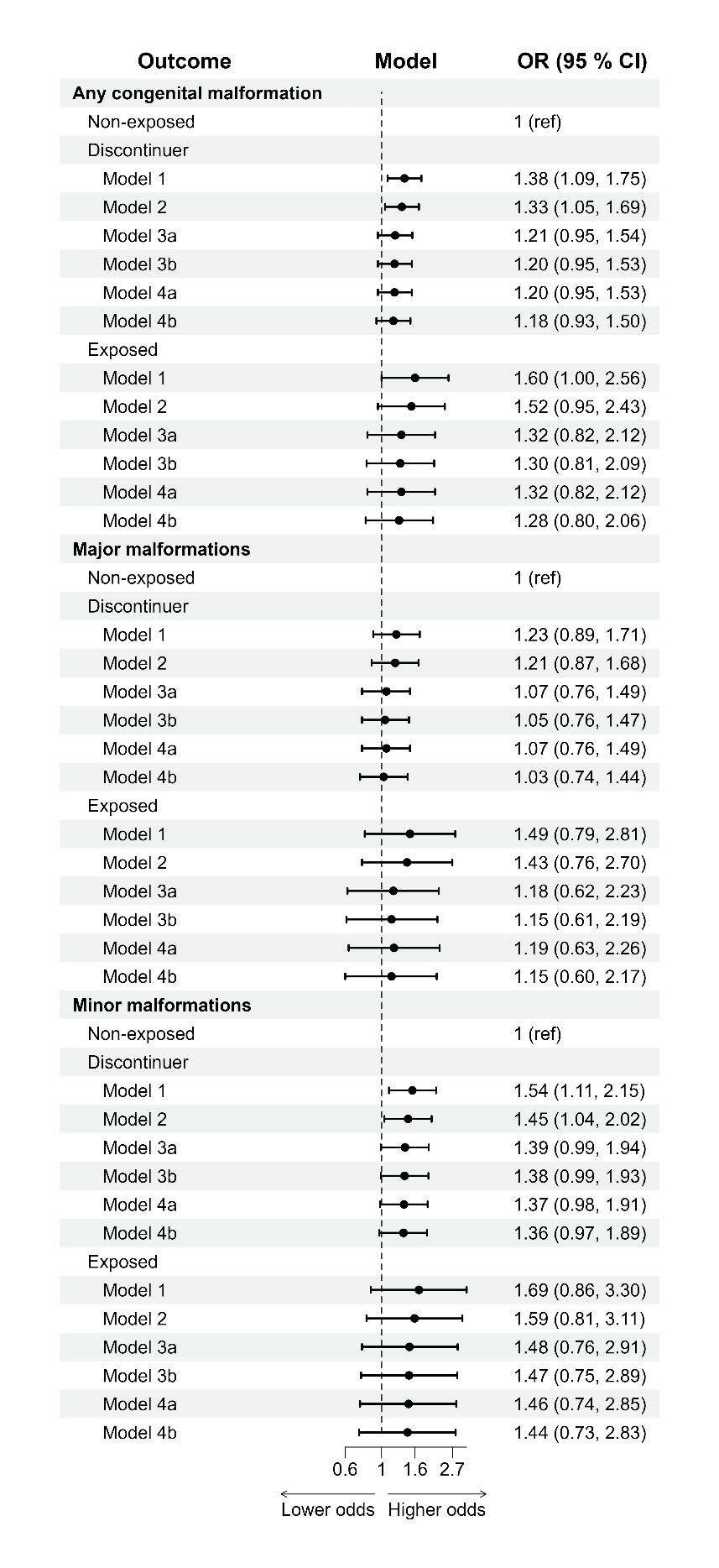
**

**Figure S3**. *Associations between statin exposure and congenital malformations, all adjustment levels.* The exposed group was defined as prescription fill in first trimester (between conception and 93 days into pregnancy); discontinuers were defined as prescription fills up to one year before conception, but not during first trimester; and non-exposed was defined as no prescription fills during first trimester and up to one year before conception. Model 1 was the crude model. Model 2 was adjusted for age, parity, pre-pregnancy folate use, and smoking in early pregnancy. Model 3a was adjusted for covariates in Model 2 plus the co-medication index. Model 3b was adjusted for covariates in Model 3a plus the co-morbidity index. Model 4a was adjusted for covariates in Model 2, plus pre-pregnancy use of medications for diabetes mellitus, hypertension, or cardiovascular disease. Model 4b was adjusted for covariates in Model 3a plus pre-pregnancy diagnoses of diabetes mellitus, hypertension, or cardiovascular disease. Model 3b was considered the main model. Abbreviations: CI, confidence interval; OR, odds ratio.

## Figure S4


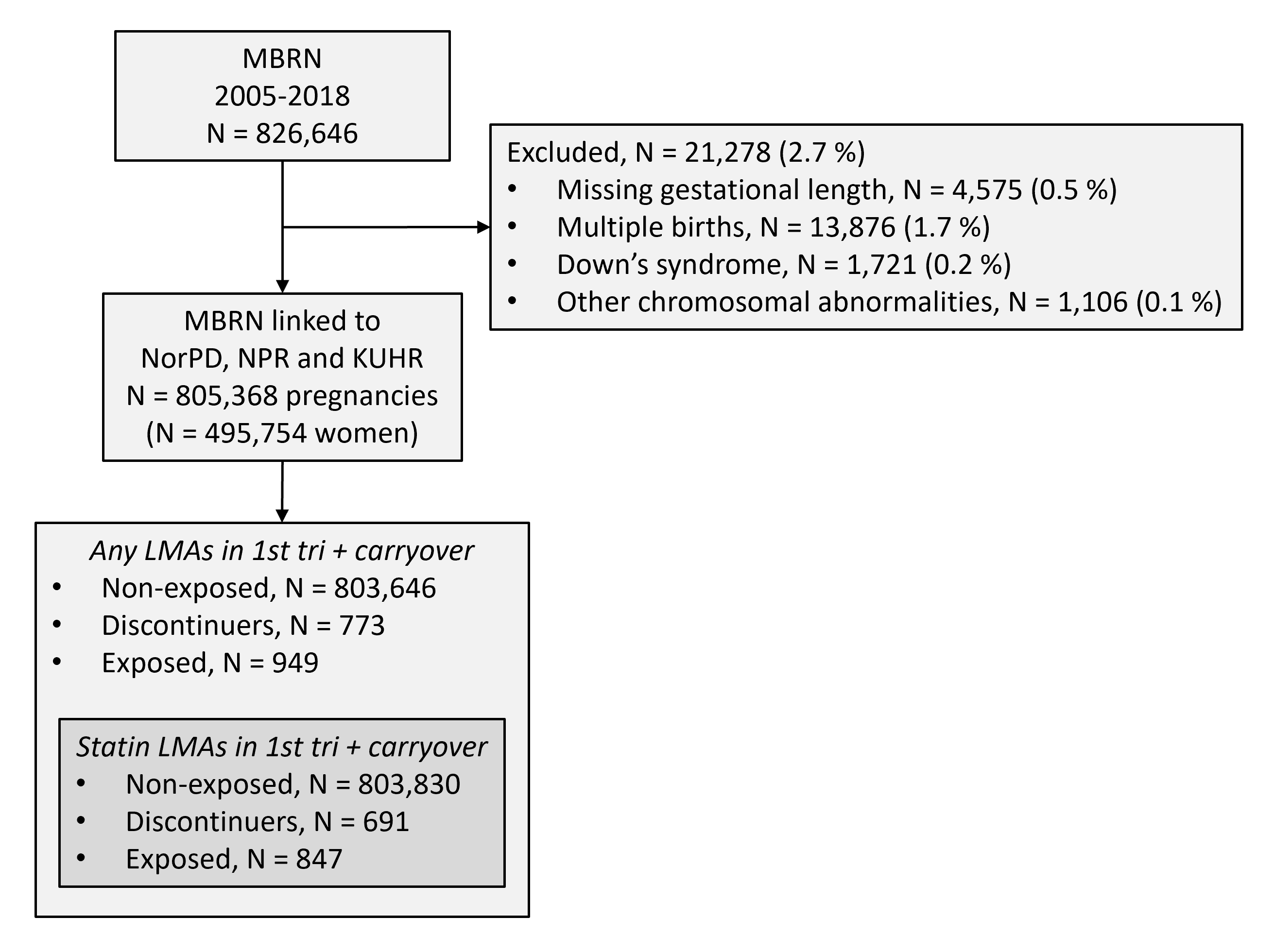


**Figure S4**. *Flow chart for carryover exposure*. We included all women with a pregnancy recorded in the MBRN in 2005-2018 and linked to NorPD, NPR and KUHR. See Methods for definitions of non-exposed, discontinuers and exposed groups, and the carryover definition. Abbreviations: KUHR, the Norway Control and Payment of Health Reimbursement Database; MBRN, the Medical Birth Registry of Norway; NorPD, the Norwegian Prescription Registry; NPR; the Norwegian Patient Registry.

## Figure S5


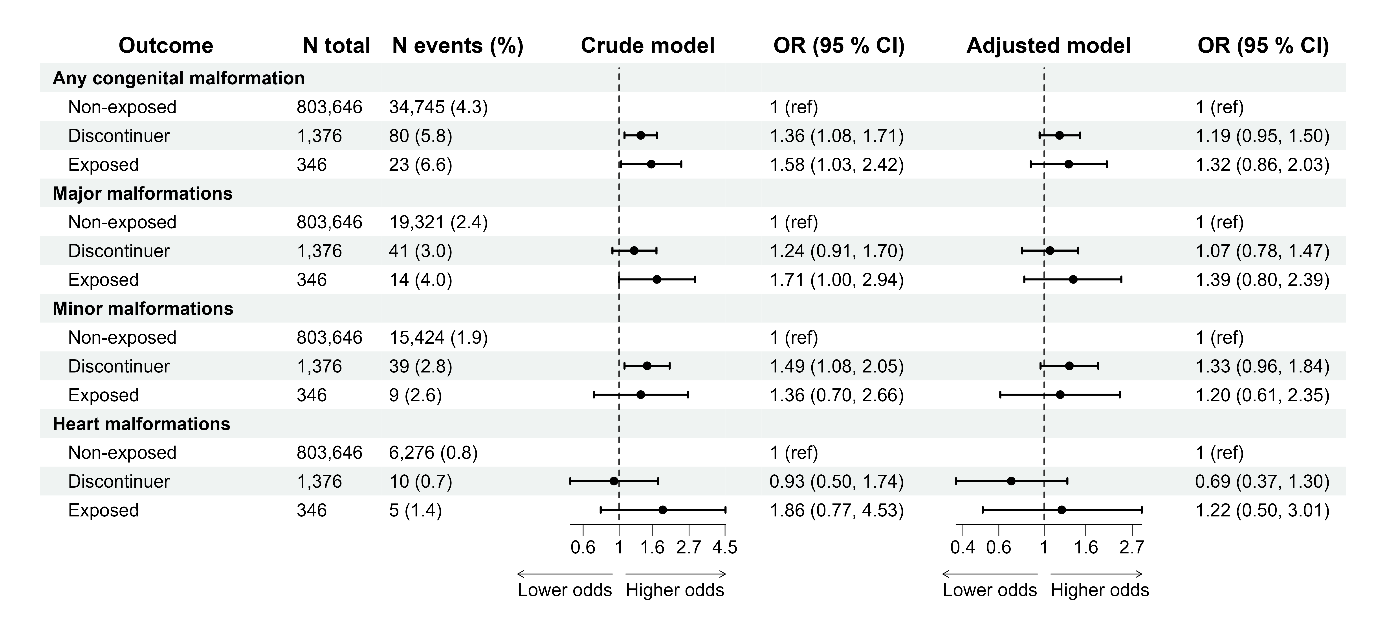


**Figure S5**. *Associations between any LMA exposure and congenital malformations*. The exposed group had prescription fills in first trimester, while discontinuers had prescription fills up to one year before conception, but not during first trimester; and non-exposed was defined as no prescription fills during first trimester and up to one year before conception. Adjustments were made for age, parity, pre-pregnancy folate use, early pregnancy smoking, comorbidity index, and comorbidity severity (co-medication) index. Abbreviations: LMA, lipid-modifying agent.

## Figure S6


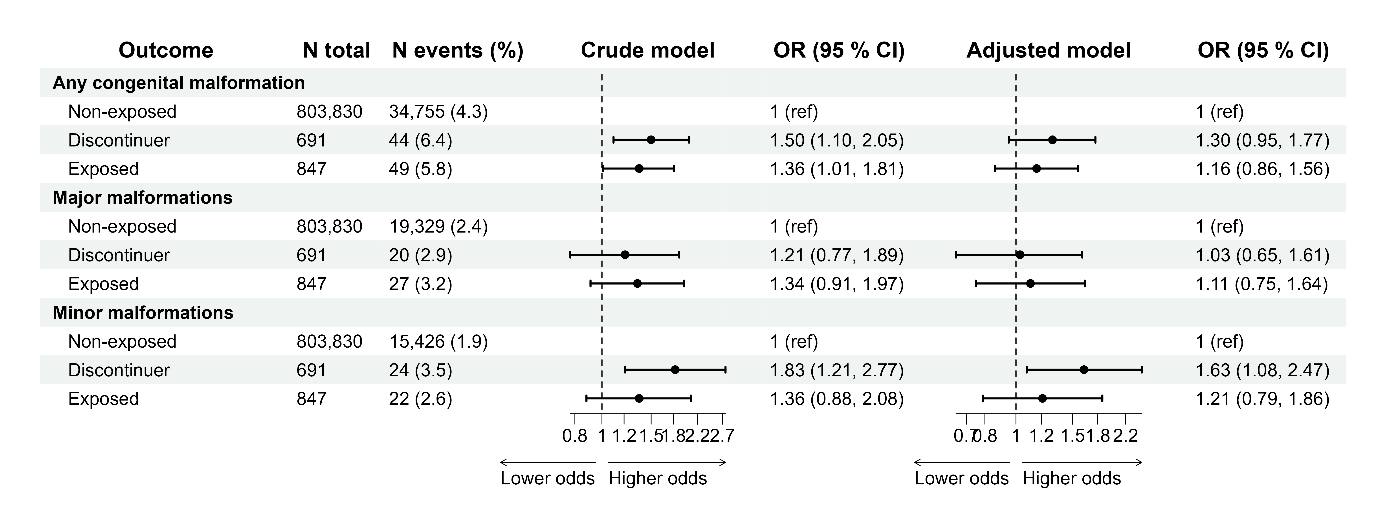


**Figure S6**. *Associations between statin carryover exposure and congenital malformations*. The exposed group had prescription fills in first trimester or before conception with carryover into first trimester, while discontinuers had prescription fills up to one year before conception, but not during first trimester or pre-conception carryover; and non-exposed had no prescription fills during first trimester and up to one year before conception. Adjustments were made for age, parity, pre-pregnancy folate use, early pregnancy smoking, comorbidity index, and comorbidity severity (co-medication) index.

## Figure S7


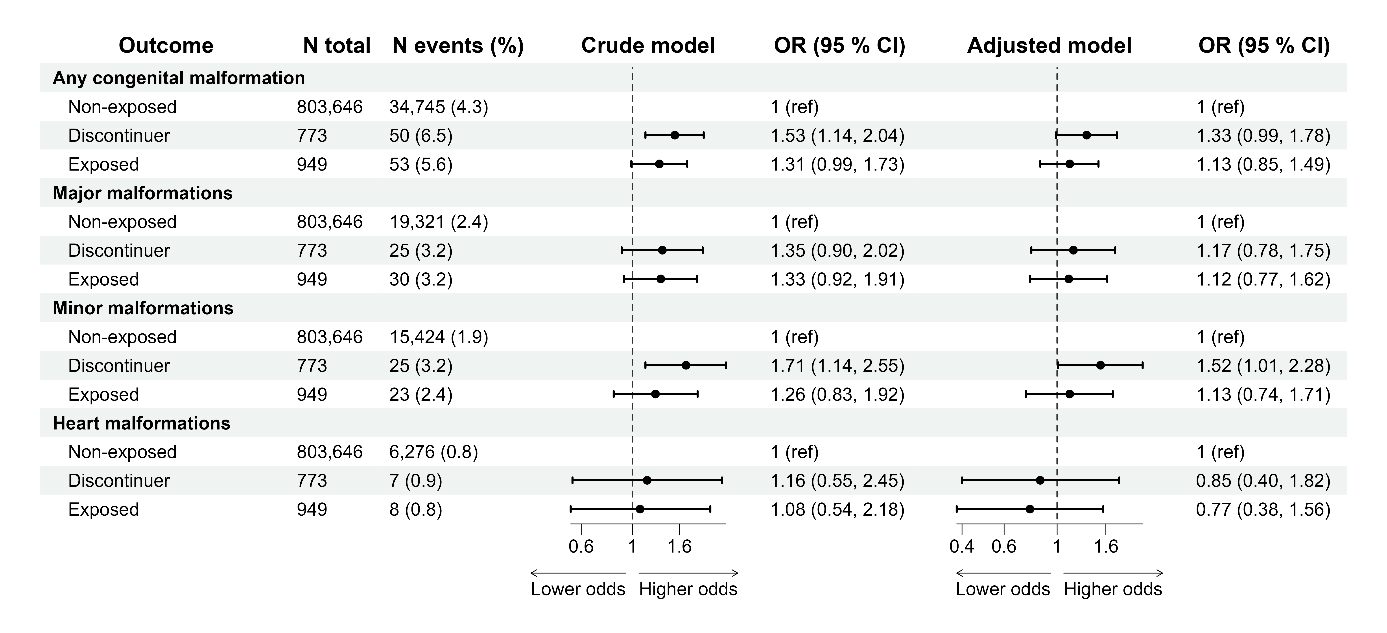


**Figure S7**. *Associations between any LMA carryover exposure and congenital malformations*. The exposed had as prescription fill in first trimester or before conception with carryover into first trimester, while discontinuers had prescription fills up to one year before conception, but not during first trimester or pre-conception carryover; and non-exposed had no prescription fills during first trimester and up to one year before conception. Adjustments were made for age, parity, pre-pregnancy folate use, early pregnancy smoking, comorbidity index, and comorbidity severity (co-medication) index. Abbreviations: LMA, lipid-modifying agent.

## Figure S8

**
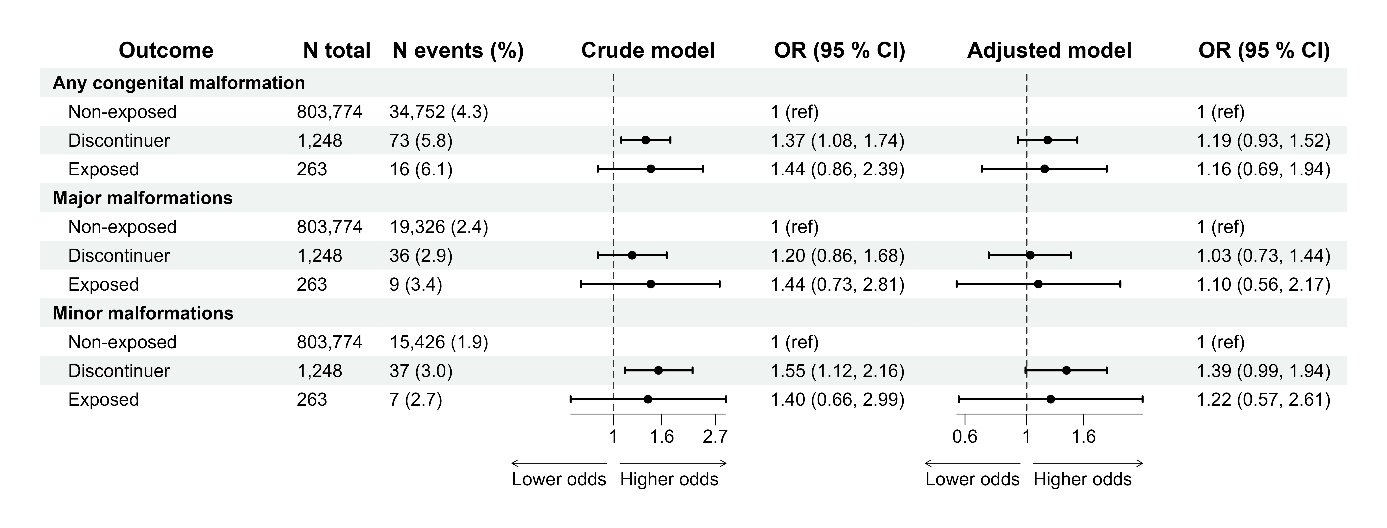
**

**Figure S8**. *Associations between statin exposure and congenital malformations, excluding pregnancies exposed to non-statin LMAs in first trimester.* The exposed group had prescription fills in first trimester, while discontinuers had prescription fills up to one year before conception, but not during first trimester; and non-exposed had no prescription fills during first trimester and up to one year before conception. Non-statin LMAs was defined as non-statin LMA monotherapy or combination therapy with statin and non-statin LMAs. Adjustments were made for age, parity, pre-pregnancy folate use, early pregnancy smoking, comorbidity index, and comorbidity severity (co-medication) index. Abbreviations: CI, confidence interval; OR, odds ratio.

## Figure S9

**
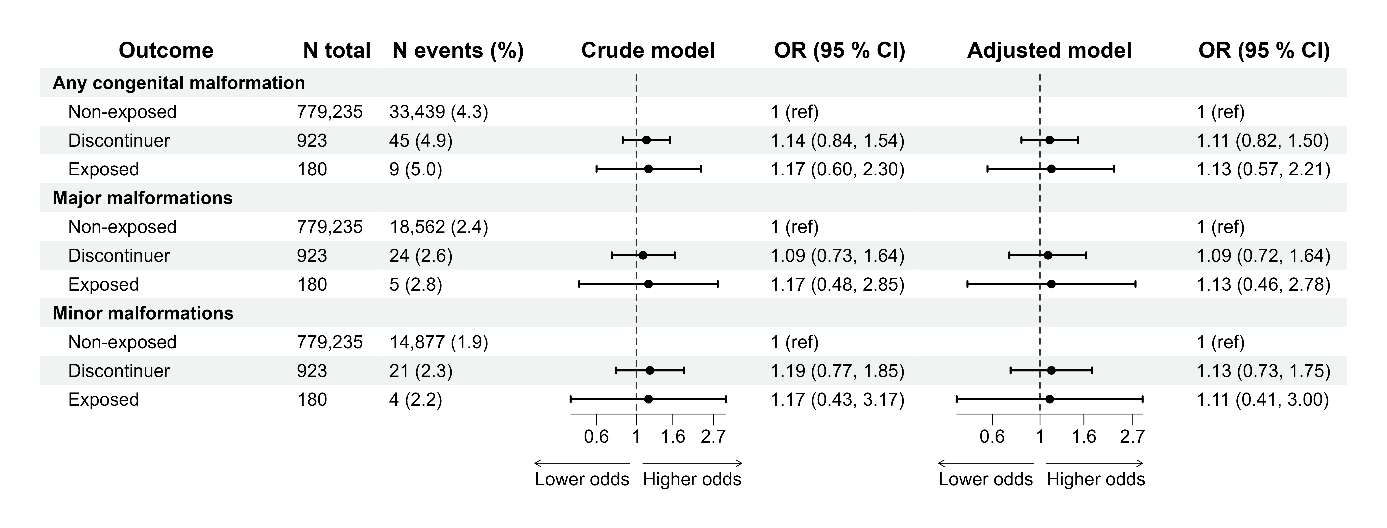
**

**Figure S9**. *Associations between statin exposure and congenital malformations, excluding pregnancies exposed to non-LMA medications in first trimester*. The exposed group had prescription fills in first trimester, while discontinuers had prescription fills up to one year before conception, but not during first trimester; and non-exposed had no prescription fills during first trimester and up to one year before conception. Non-LMA medications were defined as diabetes drugs, anti-thrombotic agents, and CVD drugs. Adjustments were made for age, parity, pre-pregnancy folate use, early pregnancy smoking, comorbidity index, and comorbidity severity (co-medication) index. Abbreviations: CI, confidence interval; OR, odds ratio.

## Figure S10

**
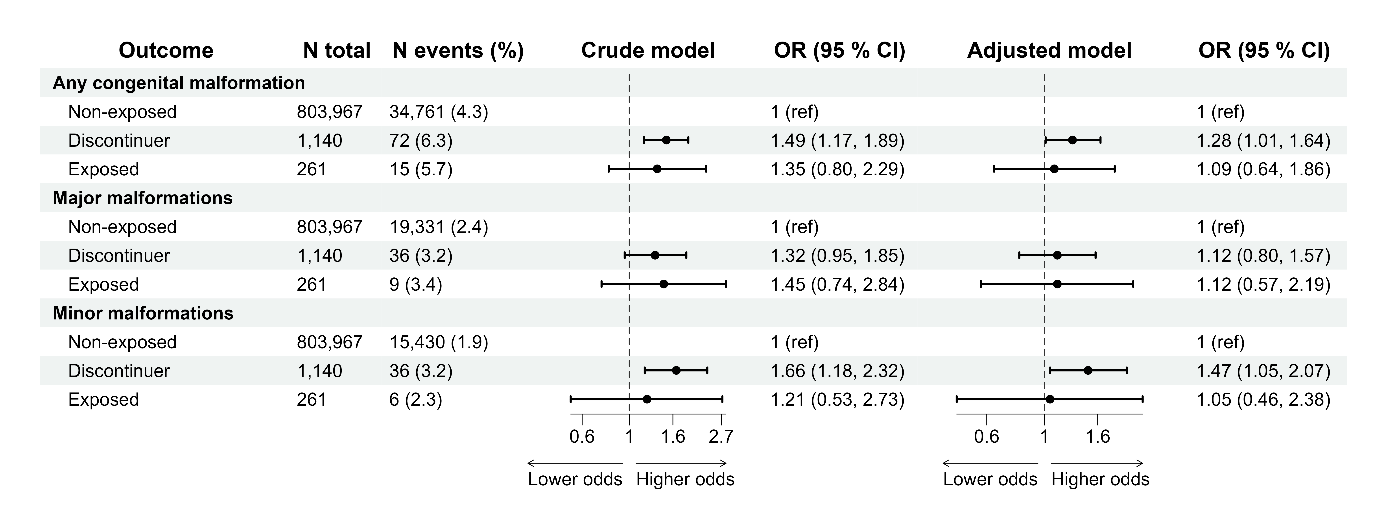
**

**Figure S10**. *Associations between lipophilic statin exposure and congenital malformations*. The exposed group had prescription fills in first trimester, while discontinuers had prescription fills up to one year before conception, but not during first trimester; and non-exposed had no prescription fills during first trimester and up to one year before conception. Lipophilic statin LMAs were defined as simvastatin, lovastatin, fluvastatin, atorvastatin, and cerivastatin. Adjustments were made for age, parity, pre-pregnancy folate use, early pregnancy smoking, comorbidity index, and comorbidity severity (co-medication) index. Abbreviations: CI, confidence interval; OR, odds ratio.

## Figure S11


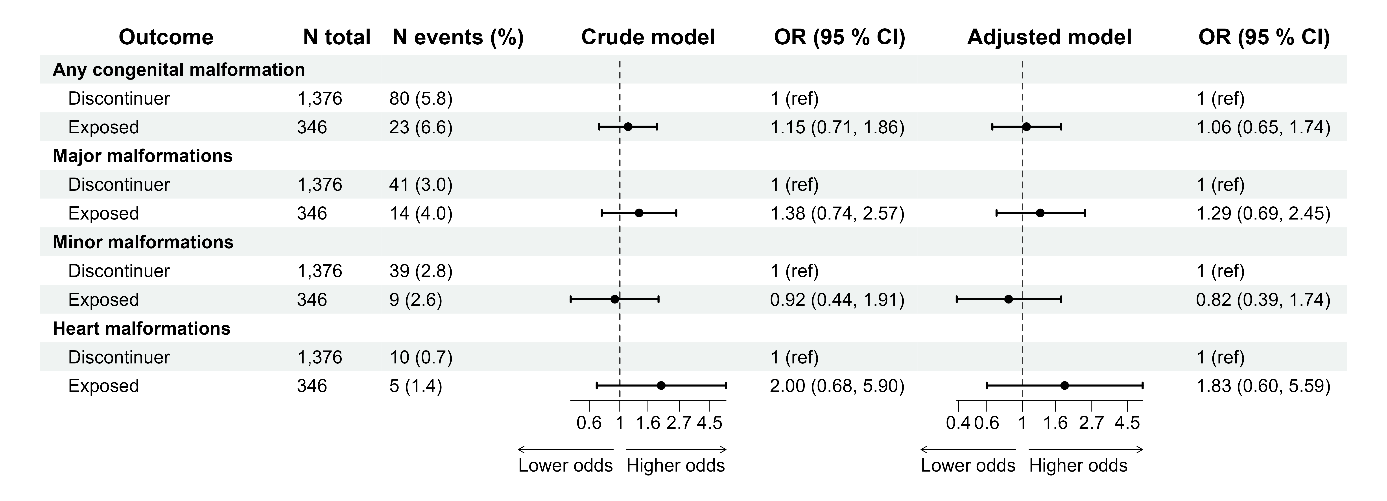


**Figure S11**. *Associations between any LMA exposure and congenital malformations among LMA continuers versus discontinuers (reference group)*. The exposed group had prescription fills in first trimester, while discontinuers were had fills up to one year before conception, but not during first trimester. Adjustments were made for age, parity, pre-pregnancy folate use, early pregnancy smoking, comorbidity index, and comorbidity severity (co-medication) index. Abbreviations: LMA, lipid-modifying agent.

## Figure S12


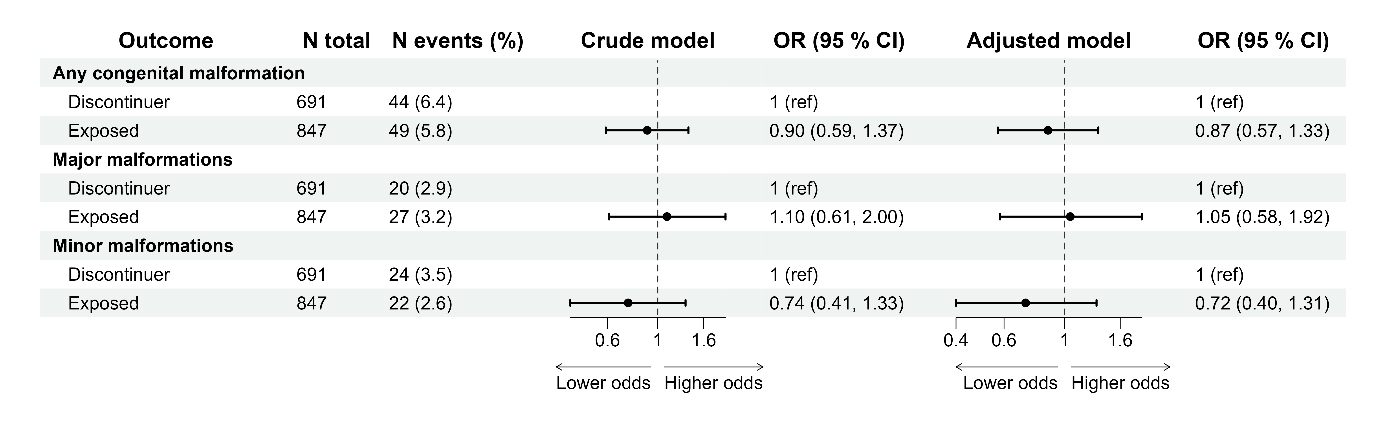


**Figure S12**. *Associations between statin carryover exposure and congenital malformations among statin continuers versus discontinuers (reference group)*. The exposed group had prescription fills in first trimester or before conception with carryover into first trimester, while discontinuers had prescription fills up to one year before conception, but not during first trimester or pre-conception carryover. Adjustments were made for age, parity, pre-pregnancy folate use, early pregnancy smoking, comorbidity index, and comorbidity severity (co-medication) index.

## Figure S13


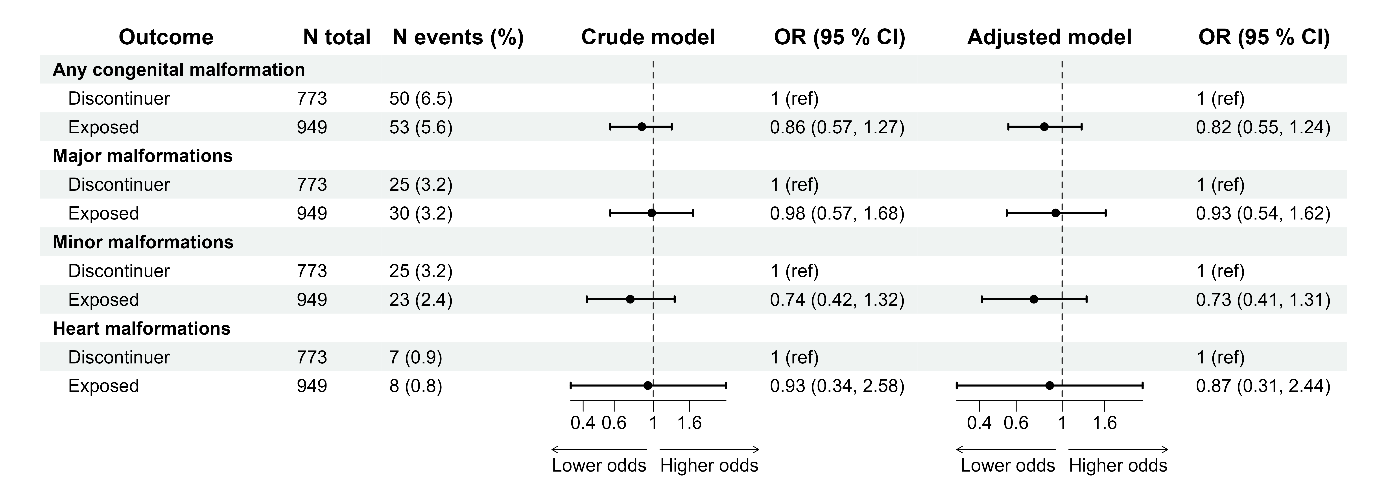


**Figure S13**. *Associations between any LMA carryover exposure and congenital malformations among LMA continuers versus discontinuers (reference group)*. The exposed group had prescription fills in first trimester or before conception with carryover into first trimester, while discontinuers had prescription fills up to one year before conception, but not during first trimester or pre-conception carryover. Adjustments were made for age, parity, pre-pregnancy folate use, early pregnancy smoking, comorbidity index, and comorbidity severity (co-medication) index. Abbreviations: LMA, lipid-modifying agent.

## Figure S14

**
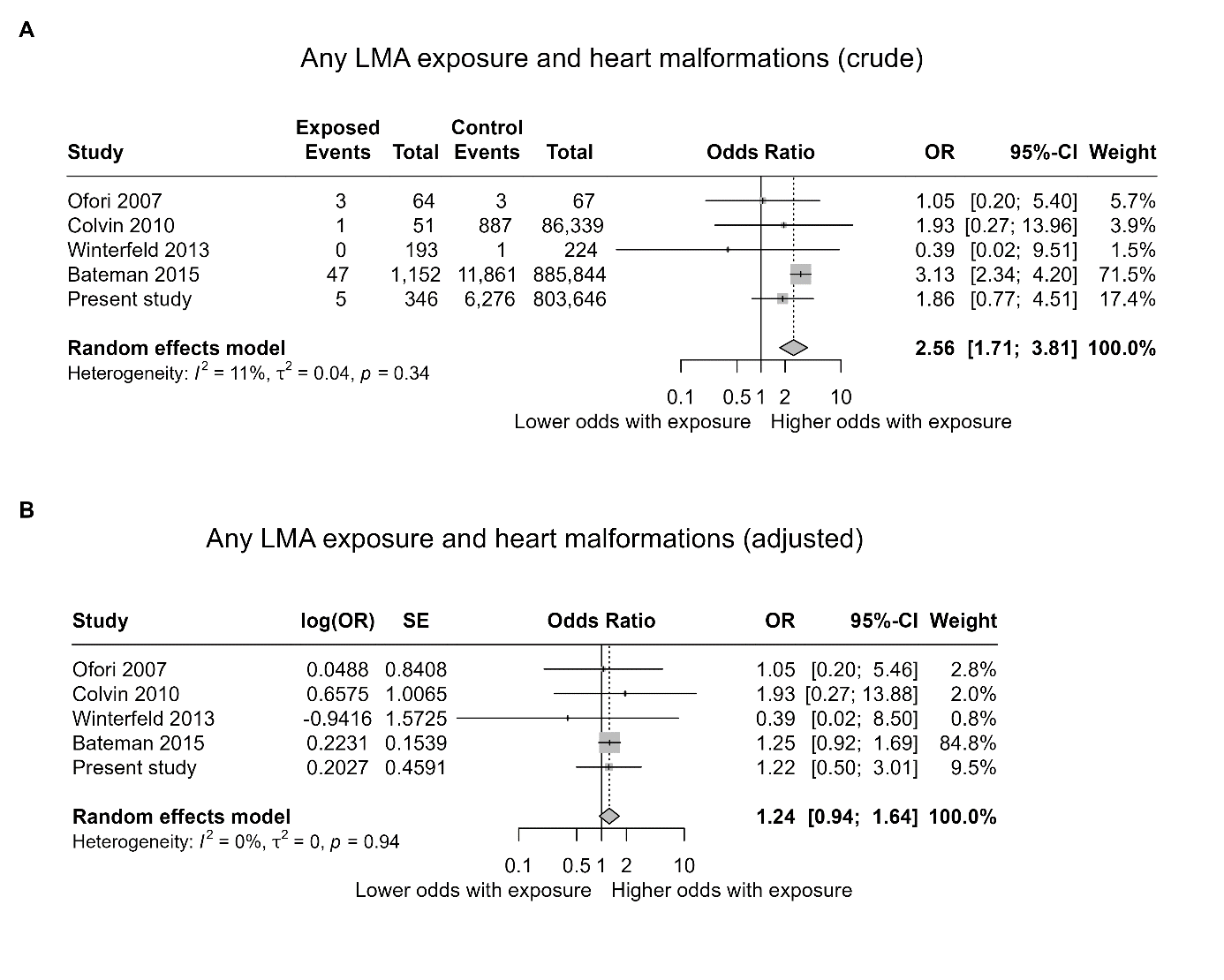
**

**Figure S14**. *Meta-analysis of studies on any LMA exposure and heart malformations.* Ofori 2007 was a nested case control study, while the other studies were cohort studies. Both crude (A) and adjusted (B) estimates are shown. Abbreviations: CI, confidence interval; I^2^, Higgins & Thompson’s I^2^ Statistic; OR, odds ratio; *p*, P value; SE, standard error; τ^2^, tau squared.
